# Modelling the influence of dimerisation sequence dissimilarities on the auxin signalling network

**DOI:** 10.1186/s12918-016-0254-7

**Published:** 2016-03-01

**Authors:** Jonathan Legrand, Jean-Benoist Léger, Stéphane Robin, Teva Vernoux, Yann Guédon

**Affiliations:** Laboratoire de Reproduction et Développement des Plantes, CNRS, INRA, ENS Lyon, UCBL, Université de Lyon, Lyon, 69364 France; CIRAD, UMR AGAP and Inria, Virtual Plants, Montpellier, 34095 France; Mathématiques et Informatique Appliquées, AgroParisTech/INRA, Paris, 75231 France

**Keywords:** *Arabidospsis thaliana*, Auxin signalling network, Transcriptional regulation, Linear regression model, Mixture model for random graphs, Plant development, Binary and valued-graph clustering

## Abstract

**Background:**

Auxin is a major phytohormone involved in many developmental processes by controlling gene expression through a network of transcriptional regulators. In *Arabidopsis thaliana*, the auxin signalling network is made of 52 potentially interacting transcriptional regulators, activating or repressing gene expression. All the possible interactions were tested in two-way yeast-2-hybrid experiments. Our objective was to characterise this auxin signalling network and to quantify the influence of the dimerisation sequence dissimilarities on the interaction between transcriptional regulators.

**Results:**

We applied model-based graph clustering methods relying on connectivity profiles between transcriptional regulators. Incorporating dimerisation sequence dissimilarities as explanatory variables, we modelled their influence on the auxin network topology using mixture of linear models for random graphs. Our results provide evidence that the network can be simplified into four groups, three of them being closely related to biological groups. We found that these groups behave differently, depending on their dimerisation sequence dissimilarities, and that the two dimerisation sub-domains might play different roles.

**Conclusions:**

We propose here the first pipeline of statistical methods combining yeast-2-hybrid data and protein sequence dissimilarities for analysing protein-protein interactions. We unveil using this pipeline of analysis the transcriptional regulator interaction modes.

**Electronic supplementary material:**

The online version of this article (doi:10.1186/s12918-016-0254-7) contains supplementary material, which is available to authorized users.

## Background

Auxin is a key signal in plant development that regulates organogenesis from embryogenesis onward. This major phytohormone achieves its morphogenetic activity notably by regulating the transcription of a large number of downstream genes. In *Arabidopsis thaliana*, the control of gene expression in response to auxin involves a complex network of 52 transcriptional regulators, consisting of 29 AUXIN/INDOLE-3-ACETIC ACID (Aux/IAA), that do not bind DNA, and 23 AUXIN RESPONSE FACTOR (ARF), which are true transcription factors (for review, see [1, 2]).

**Fig. 1:**
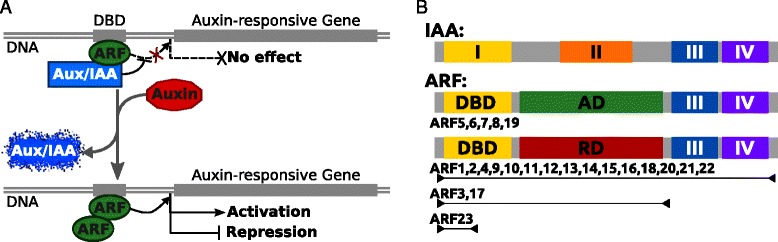
Model of auxin transduction pathway and schematic representation of the ARF and Aux/IAA structures as found in *Arabidopsis thaliana*. **a** Activation and repression activities depend on ARF middle domain amino-acid composition. DBD indicates the DNA binding domain found usptream of auxin-inducible genes. In absence of auxin, AuxIAA are dimerised with the ARF, preventing them to exert their activating or repressing activity. When auxin is present, it targets the Aux/IAA to the proteasome leaving the ARF free to dimerise and exert their regulating activity. Source: adapted from [22]. **b** DBD: DNA binding domain; I: Aux/IAA specific putative homo-dimerisation domain; AD: Activation domain; RD: Repression domain; II: Aux/IAA specific degradation domain; III & IV: protein dimerisation domains. Arrowed lines indicate the extent of each inhibiting ARF structure. Source: adapted from [2, 23] with the notable difference that we found domains III and IV when aligning full length protein sequences for ARF13

The current molecular model of the auxin signalling pathway assumes the formation of hetero-dimers between ARF and Aux/IAA in absence of auxin (Fig. 1a). According to [3] these transcriptional regulators interact through a C-terminal dimerisation domain (CTD), made of two conserved sub-sequences known as domain III (DIII) and domain IV (DIV) (Fig. 1b). ARF can bind DNA through a DNA binding domain (DBD) and act either as activators (ARF+) or repressors (ARF-) of auxin-responsive transcription (Fig. 1b) depending on the amino acid composition of the intermediate domain linking the DBD to domain III/IV (DIII/IV). It should be noted that Aux/IAA do not have a DBD and therefore are unable to regulate alone the transcription of auxin-responsive genes. When auxin accumulates in cells as a result of polar auxin transport or changes in biosynthesis, its perception targets the Aux/IAA to the proteasome [1], leading to their subsequent degradation. This subsequently releases ARF, allowing them to regulate downstream genes.

It is only recently that the topology of the Aux/IAA - ARF network was analysed extensively [4]. A yeast-2-hybrid (Y2H) [5] high-throughput approach, has allowed to test for most possible interactions between Aux/IAA and ARF proteins (with the exception of ARF15, 21 and 23, see [4] and Methods). A binary network was built from these data and a model-based graph clustering method [6] that groups proteins on the basis of their connectivity profile (i.e. similar interactors) was used to explore this network. Three clusters of proteins, that closely matched biological groups (i.e. ARF+, ARF- and Aux/IAA) [4] were identified in this way, thus demonstrating the rather stereotypical interaction properties of ARF+, ARF- and Aux/IAA (see below for more details). Here, we extended this approach to analyse the influence of the DIII/IV primary sequence dissimilarities on the likelihood of interaction between auxin transcriptional regulators. To this end, we used a recently proposed generalisation of the mixture models for random graphs that offers the possibility to deal with valued graphs and to include explanatory variables [7]. This integrative statistical model constitutes the core of our pipeline of methods for analysing the influence of sequence dissimilarities between dimerisation domains on protein-protein interactions.

## Results and discussion

A binary network is often easier to interpret than a valued one. However, in our case, it does not fully represent the “true” biochemical network as an interaction network depends on several properties, such as interaction strength, protein concentration, spatial expression and synthesis/degradation dynamics of the proteins. We will first briefly recall how the binary network was built and analysed in [4]. Then we will compare this previous approach with the analysis of a valued network, built to minimize the loss of information, before investigating how dissimilarities between dimerisation domains can be incorporated in such a modelling framework.

### Available Y2H experimental data and binary Aux/IAA - ARF network

We used in this work a previously available Y2H dataset where Aux/IAA and ARF interactions have been tested in yeast both ways [4]. Interactions were tested for each protein fused to the activation (AD) or to the binding domain (BD) of the Gal4 yeast transcription factor, thus allowing to minimize false positives. In addition and to minimize false negatives, two reporter genes, HIS3 and X-Gal, were used for testing the interaction. In this experiment the interaction capacities of 49 transcriptional regulators were tested (ARF15, 21 and 23 could not be cloned), thus making a total of 2401 interactions tested. We give in Table 1 an example of the results. Note that the Y2H dataset was obtained using only DIII/IV for ARF, while full-length proteins were used for Aux/IAA (see Conclusions for a discussion of that point).

**Table 1:** Example of Y2H data, with the name of the tested proteins, the side they were attached to and the output returned by each reporter gene

Bait(BD)	Prey(AD)	X-Gal	HIS3	Bait(BD)	Prey(AD)	X-Gal	HIS3
BD-ARF1	AD-ARF1	−	12 *%*				
BD-ARF2	AD-ARF1	−	14 *%*	BD-ARF1	AD-ARF2	−	14 *%*
BD-ARF3	AD-ARF1	−	15 *%*	BD-ARF1	AD-ARF3	−	13 *%*
⋮	⋮	⋮	⋮	⋮	⋮	⋮	⋮
BD-IAA2	AD-ARF5	++	90 *%*	BD-ARF5	AD-IAA2	+?	119 *%*
BD-IAA3	AD-ARF5	++	90 *%*	BD-ARF5	AD-IAA3	++	121 *%*
BD-IAA4	AD-ARF5	++	121 *%*	BD-ARF5	AD-IAA4	+++	70 *%*
⋮	⋮	⋮	⋮	⋮	⋮	⋮	⋮

**Fig. 2:**
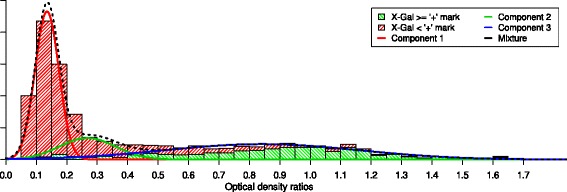
Three-component Gaussian mixture model estimated on the basis of the optical density (OD) ratio sample (HIS3 test). OD ratios are represented up to a limit of 1.7 for readability reasons

The Y2H data were previously used to build a binary network [4]. This required choosing thresholds for both tests on the basis of their empirical distributions. The threshold was set between the successive marks ‘+?’ and ‘+’ for the X-Gal test (see Methods for detailed explanations) and at 0.45 for the HIS3 test [4]; see an illustration of these thresholds in Fig. 2. Decision rules were then used to combine the four test outputs ([4]; see Methods).

We aimed at analysing binary and valued networks potentially influenced by dimerisation sequence dissimilarities. These networks should be built on the same transcriptional regulators. We therefore excluded ARF11 since this ARF showed no interactions in the previously published Aux/IAA - ARF binary network [4]. We also excluded ARF3 and 17 since they do not possess DIII/IV. We then built a new binary network using the same thresholds as in [4]. We also tested HIS3 thresholds at 0.3 and 0.65. Applying these thresholds only slightly modify the binary network (Additional file 1: Figure S2). The binary network built using the HIS3 threshold at 0.45 will thus be used in the rest of this work.

### Building a valued network from Y2H data

Combining the X-Gal and HIS3 test outputs in a single interaction distance requires a standardization procedure (see Methods). The objective of standardization is to avoid dependency on the elementary distance type and scale. It is important to point out that, in our case, the valued network does not represent affinities between proteins, but rather the likelihoods of interaction between proteins. We tested several weightings of the outputs of the X-Gal and HIS3 tests and in particular:

- network **A**: *w*_X-Gal_=0.75 and *w*_HIS3_=0.25;
- network **B**: *w*_X-Gal_=0.5 and *w*_HIS3_=0.5;
- network **C**: *w*_X-Gal_=0.25 and *w*_HIS3_=0.75.

**Fig. 3:**
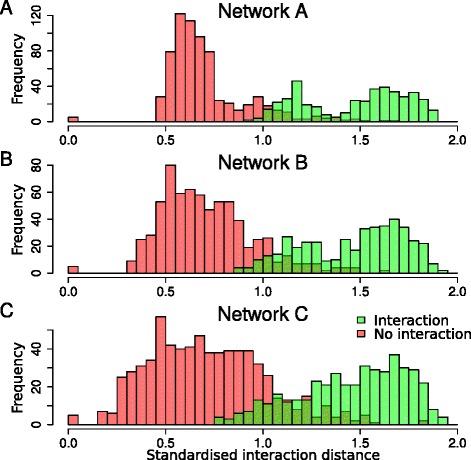
Empirical distribution of the standardised interaction distances for the three valued networks. **a** Network A: *w*_X-Gal_=0.75 and *w*_HIS3_=0.25. **b** network B: *w*_X-Gal_=0.5 and *w*_HIS3_=0.5. **c** network C: *w*_X-Gal_=0.25 and *w*_HIS3_=0.75. The presence and absence of interaction, as identified in the presented binary network, are represented respectively in green and red

To this end, we visualized the standardised distance distributions corresponding to “no interaction” (red) and “interaction” (green) according to the previously defined binary assignment (Fig. 3). Network C is characterized by standardised distances corresponding to “no interaction” spread over a wide range of values, thus leading to a rather large overlap with standardised distances corresponding to “interaction”. Network A on the contrary concentrates standardised distances corresponding to “no interaction” over a small range of values, leading to a clear separation with standardised distances corresponding to “interaction”. Finally, network B (corresponding to the balanced weighting) presents a reasonable compromise between the dispersion of standardised distances corresponding to “no interaction” and “interaction” and their overlap. This comparison of the networks highlights the fact that the X-Gal test seems more reliable than the HIS3 test in this dataset, probably because of the very long tail corresponding to higher interaction likelihoods for this test (Fig. 2). In the following, we will thus present clustering results only for networks A and B.

### Network topology analysis using Bernoulli and Gaussian mixture models

To gain further insights into the binary and valued networks topology, we applied a model-based graph clustering methods in order to group the transcriptional regulators on the basis of their connectivity profiles. The key feature of mixture models for random graphs is to give a probabilistic summary of the connectivity structure by uncovering clusters of proteins that share similar connectivity profiles. The parameters of the model are the cluster weight distribution and the connectivity distributions for each pair of clusters.

In the case of a binary adjacency matrix *Z*, connectivity distributions are Bernoulli distributions parametrized by connectivity probabilities, that is the probability for proteins of two clusters to interact:

(1) $$ Z_{ij}|\left\{i\in C_{q},j\in C_{\ell}\right\} \sim\mathcal{B}(\pi_{\textit{q}\ell}).   $$

The interaction *Z*_*ij*_ between vertices *i* and *j* knowing that *i* belongs to cluster *q* and *j* to cluster *ℓ* follows a Bernoulli distribution of parameter *π*_*q*,*ℓ*_.

In the case of a weighted adjacency matrix *X*, the connectivity distributions are Gaussian distributions:

(2) $$ X_{ij}|\left\{i\in C_{q},j\in C_{\ell}\right\} \sim\mathcal{N}\left(\mu_{\textit{q}\ell},\sigma^{2}\right).   $$

It should be noted that parameter *μ*_*qℓ*_ of a Gaussian mixture (GM) model is the mean likelihood of interaction between proteins of two clusters. This is different from the Bernoulli mixture (BM) model where the parameter *π*_*qℓ*_ is the probability for proteins of two clusters to interact. This makes the biological interpretation of GM model parameters less straightforward.

The inference of such models is not restricted to the estimation of the cluster weight and connectivity distributions but encompasses the inference of the number of clusters using a penalized likelihood criterion. The principle of penalized likelihood criteria such as the integrated completed likelihood (ICL) criterion consists in making a trade-off between an adequate fitting of the model to the data and a reasonable number of parameters to be estimated. The ICL criterion is specifically tailored to the clustering objective and is expected to favour models such that the uncertainty of protein assignment to clusters is low. Jeffreys’ rules of thumb [8] suggest that a difference of ICL of at least log(100)=4.6 is needed to deem the model with the higher ICL substantially better. Since the ICL criterion is only asymptotically valid (i.e. for large *N*), the number of clusters given by this criterion should be considered as indicative. We thus chose to systematically investigate potential interesting clusterings combining ICL values, prior biological knowledge and within- and between-cluster distances for assessing the homogeneities and separabilities of clusters. One key output for the validation of a model-based clustering method is the posterior distributions of protein assignment to clusters. For each protein, this posterior distribution was degenerate (probability of 1 for a given cluster and 0 for the others) whatever the model used, which eased the interpretation of the clustering outputs.

#### Building a Bernoulli mixture model

Note that the clustering results reported here using BM models slightly differ from those presented in [4] since we only used 46 proteins (instead of 49 proteins, as explained above).

When estimating BM models on the basis of the 46 protein binary network, the ICL criterion favours first the 6-cluster BM model and next the 4-cluster BM model (Table 2). However, the ICL difference (*Δ*ICL<2) between the 4- and the 6-cluster BM models was not significant according to Jeffreys’ rules of thumb.

**Table 2:** ICL criterion values and corresponding posterior model probabilities for BM, GM-A and GM-B models

	No. clusters	1	2	3	4	5	6	7
BM	ICL	−	−527.3548	−521.8064	−506.7471	−511.0779	−504.9562	−507.8915
	post. proba.	−	0	0	0.136	0.002	0.818	0.043
GM-A	ICL	−595.221	−333.666	−283.778	−268.434	−258.972	−260.468	−268.91
	post. proba.	0	0	0	0	0.817	0.183	0
GM-B	ICL	−617.343	−344.357	−306.136	−286.626	−279.985	−265.725	−278.627
	post. proba.	0	0	0	0	0	1	0

**Fig. 4:**
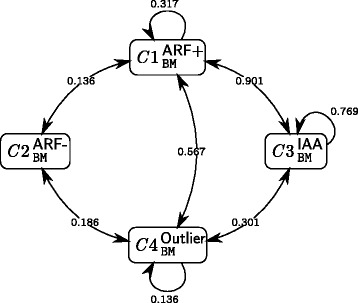
Connectivity graph and associated probabilities for the 4-cluster BM model. The connectivity matrix describes the topology of the network at the cluster scale. The *π*_*qℓ*_ values are the probability for a protein of cluster *q* to interact with a protein of cluster *ℓ*. Only probabilities above 0.1 are represented

For the 4-cluster BM model (Table 3), we found three clusters corresponding to biologically meaningful groups and an “outlier” cluster. The three clusters $C1^{\text {ARF+}}_{\texttt {BM}}$, $C2^{\text {ARF-}}_{\texttt {BM}}$ and $C3^{\text {IAA}}_{\texttt {BM}}$ show a specific enrichment in respectively ARF+, ARF- and Aux/IAA. The fourth cluster $C4^{\text {Outlier}}_{\texttt {BM}}$ can be categorized as “outlier” since it groups one ARF- with six Aux/IAA in addition of being poorly defined as detailed below. A connectivity graph representing the interaction probability between clusters is given in Fig. 4.

**Table 3:** Composition of the four clusters obtained using the BM model

$C1^{\text {ARF+}}_{\texttt {BM}}$	**ARF5** (0.19), **ARF19** (0.212), **ARF8**, **ARF7**, **ARF6** (0.258), IAA5 (0.299), ARF9, IAA9, IAA34
$C2^{\text {ARF-}}_{\texttt {BM}}$	ARF14 (0.087), ARF1 (0.096), ARF13, ARF16 (0.115), IAA6, ARF4, ARF10, ARF18, ARF2 (0.137), ARF12 (0.154), ARF20 (0.189)
$C3^{\text {IAA}}_{\texttt {BM}}$	IAA3 (0.198), IAA8 (0.205), IAA4 (0.222), IAA2, IAA18, IAA1, IAA16, IAA28 (0.25), IAA15 (0.261), IAA10, IAA12, IAA13, IAA27, IAA19 (0.284),
	IAA14 (0.296), IAA17, IAA20 (0.307), IAA30 (0.33), IAA7
$C4^{\text {Outlier}}_{\texttt {BM}}$	IAA11 (0.333), ARF22 (0.337), IAA26, IAA29 (0.348), IAA33 (0.377), IAA32, IAA31 (0.435)

The ARF activators are in bold. The distance *D*(*i, q*) between protein *i* and cluster *q* to which it is assigned is given for the most central, the most peripheral and some other proteins of interest for interpretation

An important criterion to assess the validity of a clustering model is the between-cluster distance matrix *D*(*q*,*ℓ*) (given below). $C1^{\text {ARF+}}_{\texttt {BM}}$, $C2^{\text {ARF-}}_{\texttt {BM}}$ and $C3^{\text {IAA}}_{\texttt {BM}}$ present within-cluster distances (diagonal) smaller than between-cluster distances (off diagonal), showing a strong definition of these clusters (see Eq. 3). The within-cluster distance of $C4^{\text {Outlier}}_{\texttt {BM}}$ is in contrast higher than the within-cluster distance of the three other clusters. In addition, its within-cluster distance is larger that its distance to $C2^{\text {ARF-}}_{\texttt {BM}}$. This configuration can be interpreted in the framework of density-based clustering (see [9] and references therein) where $C1^{\text {ARF+}}_{\texttt {BM}}$, $C2^{\text {ARF-}}_{\texttt {BM}}$ and $C3^{\text {IAA}}_{\texttt {BM}}$ are characterized by rather high density of elements with respect to the density of elements of $C4^{\text {Outlier}}_{\texttt {BM}}$. This outlier cluster might be explained in part by biological noise in the Y2H experiments.

(3) $${} \begin{aligned} &\hspace{60pt} C1^{\text{ARF+}}_{\texttt{BM}} \hspace{5pt} C2^{\text{ARF-}}_{\texttt{BM}} \hspace{5pt} C3^{\text{IAA}}_{\texttt{BM}} \hspace{5pt} C4^{\text{Outlier}}_{\texttt{BM}}\\ &D_{\text{BM}}(q,\ell) = \left(\begin{array}{cccc} 0.257 & \quad0.533 & \quad0.364 & \quad0.512\\ 0.533 & \quad0.124 & \quad0.524 & \quad0.314\\ 0.364 & \quad0.524 & \quad0.260 & \quad0.435\\ 0.512 & \quad0.314 & \quad0.435 & \quad0.354 \end{array}\right)\\ \end{aligned}  $$

In the case of the 6-cluster BM model favoured by the ICL criterion, two clusters are not well defined in terms of within- and between-cluster distances (Additional file 2: Table S1). The cluster composition shows three Aux/IAA enriched clusters and one outlier cluster (compare the 4- and 6-cluster BM models cluster composition in Additional file 1: Figure S3). This is likely a consequence of the tendency of the ICL criterion to select overparameterized models in our context.

Taken together these results suggest that the 4-cluster BM model is more relevant both from the point of view of cluster definition and biological meaning. As we will see later, a clustering with three biologically meaningful clusters and an “outlier” cluster is supported by the different models and will therefore be used for comparing the outcome of these models.

#### Building Gaussian mixture models

We next used GM models for analysing the A and B valued networks. The ICL criterion favours the 5-cluster GM model for network A and the 6-cluster GM model for network B (Table 2). The more parsimonious model selected for network A may be due to the high dispersion of HIS3 values which have less weight in network A than in network B (*w*_HIS3_=0.25 for network A and *w*_HIS3_=0.5 for network B) (Fig. 2). This supports the idea that the X-Gal test is more reliable than the HIS3 test. We thus chose to focus on GM models built on the basis of network A (GM-A model).

Analysing the cluster composition for the 5-cluster GM-A model, we found three biologically meaningful and two “outlier” clusters (see Additional file 1: Figure S4B for the cluster composition). When assessing the clustering quality, we observed that the third cluster, specifically enriched in Aux/IAA, presented a rather large within-cluster distance compared to its distances to the other clusters (see Additional file 2: Table S2). The two “outliers” clusters not being that well defined too, we decided to compare the 5-cluster GM-A model with the 4-cluster GM-A model since it corresponds to the most relevant clustering found using BM models.

This 4-cluster GM-A model exhibits a meaningful biological structure with three clusters *C*1GM-AARF+,*C*2GM-AARF- and $C3^{\text {IAA}}_{\texttt {GM-A}}$ specifically enriched in each family of proteins and an “outlier” cluster $C4^{\text {Outlier}}_{\texttt {GM-A}}$. Remarkably, $C4^{\text {Outlier}}_{\texttt {GM-A}}$ is the merging of the two “outlier” clusters identified with the 5-cluster GM-A model with the exception of IAA29 found in $C2^{\text {ARF-}}_{\texttt {GM-A}}$ for the 4-cluster GM-A model (see the compositions in Additional file 1: Figure S4A and B). Thus, when assessing clustering on the basis of the cluster-distance matrix (see Eq. 4) we still observe a rather large within-cluster distance for $C3^{\text {IAA}}_{\texttt {GM-A}}$ compared to its distances to the other clusters. Since the 5-cluster model favoured by the ICL criterion is almost perfectly nested in the 4-cluster model, we argue here that the simpler model is more relevant. Again, this can be interpreted as the tendency of the ICL criterion to select overparameterized models.

(4) $${} \begin{aligned} &\hspace{70pt} C1^{\text{ARF+}}_{\texttt{GM-A}} \hspace{5pt} C2^{\text{ARF-}}_{\texttt{GM-A}} \hspace{5pt} C3^{\text{IAA}}_{\texttt{GM-A}} \hspace{5pt} C4^{\text{Outlier}}_{\texttt{GM-A}}\\ &D_{\text{GM-A}}(q,l) = \left(\!\!\begin{array}{cccc} {\phantom{1}}0.015 & \quad0.016 & \quad0.032 & \quad0.024{\phantom{1}}\\ {\phantom{1}}0.016 & \quad0.013 & \quad0.016 & \quad0.016{\phantom{1}}\\ {\phantom{1}}0.032 & \quad0.016 & \quad0.032 & \quad0.022{\phantom{1}}\\ {\phantom{1}}0.024 & \quad0.016 & \quad0.022 & \quad0.022{\phantom{1}}\\ \end{array}\!\right)\\ \end{aligned}   $$

**Fig. 5:**
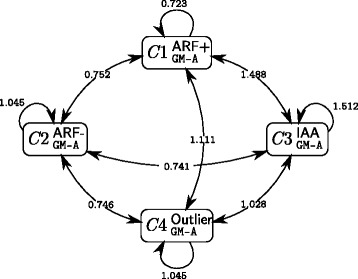
Connectivity graph and associated mean interaction likelihoods for the 4-cluster GM-A model. The connectivity matrix describes the topology of the network at the cluster scale. The values are the mean likelihoods of interaction *μ*_*q*,*ℓ*_ between a protein of cluster *q* and a protein of cluster *ℓ*

We give in Fig. 5 the connectivity graph obtained using the 4-cluster GM-A model. We stress here that the mean interaction likelihood (*μ*_*qℓ*_) should not be directly compared to the interaction probabilities (*π*_*qℓ*_) represented in the connectivity graph obtained using the BM model (Fig. 4) since they do not represent the same information; see Additional file 1: Figure S5 for the clustered valued adjacency matrix with proteins sorted by increasing within-cluster distances.

One should note a specificity of $C3^{\text {IAA}}_{\texttt {GM-A}}$ in Table 4 whose lowest protein to cluster distance (0.028) is greater than the highest protein to cluster distances (0.019, 0.016, 0.024) for the three other clusters. This explain the large within-cluster distance observed for $C3^{\text {IAA}}_{\texttt {GM-A}}$; see Eq. 4.

**Table 4:** Composition of the four clusters obtained using the GM-A model

$C1^{\text {ARF+}}_{\texttt {GM-A}}$	**ARF5** (0.01), **ARF7**, **ARF8**, IAA9, **ARF19**, **ARF6** (0.019)
$C2^{\text {ARF-}}_{\texttt {GM-A}}$	ARF1 (0.012), ARF10, IAA6, IAA11, ARF4 (0.013), ARF14, ARF16, ARF18, , ARF20 (0.014), ARF12 (0.015), ARF13, ARF2 (0.016)
$C3^{\text {IAA}}_{\texttt {GM-A}}$	IAA15 (0.028), IAA10, IAA31, IAA2, IAA14, IAA1 (0.031), IAA12, IAA18, IAA4 (0.033), IAA17, IAA27, IAA19 (0.034), IAA3, IAA8, IAA16, IAA28,
	IAA34, IAA5 (0.036)
$C4^{\text {Outlier}}_{\texttt {GM-A}}$	(0.019), (0.024), (0.024)

The ARF activators are in bold. The proteins that are assigned to the two outlier clusters in the 5-cluster GM-A model are respectively in blue and cyan. The distance *D*(*i, q*) between protein *i* and its cluster *q* is given for the most central, the most peripheral and some other proteins of interest for interpretation. See Additional file 1: Figure S4A for the distance plot

#### Comparing Bernoulli and Gaussian mixture model clusterings

Cluster compositions of the 4-cluster BM model (Table 3) and GM-A model (Table 4) are rather similar with 78 *%* match (Table 5). The differences in cluster assignment concern almost only peripheral elements of the clusters and the core of the four clusters are very similar.

**Table 5:** Cluster composition matching for the 4-cluster models (percentage and number of matches): Bernoulli mixture (BM) model, Gaussian mixture models based on networks A (GM-A) and B (GM-B), linear regression mixture models with a single (LRM-1) and two explanatory variables (LRM-2)

Models	BM	GM-A	GM-B	LRM-1	LRM-2
BM	100 % (46)	78 % (36)	74 % (34)	76 % (35)	76 % (35)
GM-A	.	100 % (46)	96 % (44)	87 % (40)	91 % (42)
GM-B	.	.	100 % (46)	87 % (40)	91 % (42)
LRM-1	.	.	.	100 % (46)	96 % (44)
LRM-2	.	.	.	.	100 % (46)

The between-cluster distance matrices suggest that the BM model performs better than the GM-A model, allowing for a better definition of the clusters according to within- and between-cluster distances. While it may introduce errors (false positives or negatives) depending on the thresholds and decision rules defined for the X-Gal and HIS3 tests, the binarisation of interactions is thus likely to effectively remove experimental noise. On the opposite, the standardization is a more objective approach, since it scales the outputs of the X-Gal and HIS3 tests to make them comparable and limits the loss of information. However, standardization does not remove experimental noise, which seems to be in our case a shortcoming for cluster definition. Nevertheless, with both BM and GM models, we were able to identify a strong core structure in the auxin signalling network, closely related to the predicted biological structure [3].

### Analysing the influence of the protein primary sequence dissimilarities on the auxin network topology using linear regression mixture models

We next sought to address how the evolution of multigenic families such as the one encoding ARF and Aux/IAA has influenced the auxin signalling network topology by modifying the dimerisation capacities of its members. To get insights into this complex question, we ask here whether dissimilarities in primary sequences of ARF and Aux/IAA dimerisation domain influence the topology of the Aux/IAA - ARF network. Note that we only present results for network A and use the distance between primary sequences as a measure of protein dissimilarities.

#### Building the dimerisation domain protein distance matrix

One way to analyse the influence of DIII/IV primary sequence on the Aux/IAA - ARF network is to incorporate distances between protein sequences as explanatory variables in a linear regression mixture (LRM) model. To build a distance matrix corresponding to the dimerisation domain differences in terms of amino acid se- quences, we started by aligning full protein sequences of all Aux/IAA and ARF presenting the conserved CTD domain (DIII/IV) using CLUSTAL-W [10]. To recover DIII and DIV amino acid sub-sequences, we searched for conserved patterns among the aligned sequences using Gblocks [11]. Two conserved blocks were found at the C-terminal part of the sequences, corresponding to the two sub-domains DIII and DIV (Methods and Additional file 1: Figure S6). The per-site protein distance matrix was then obtained using the amino acid substitution model PAM computed with PROTDIST. We also computed two distance matrices, corresponding respectively to DIII and DIV, to be used in LRM models with two explanatory variables (see below).

#### Linear regression mixture models with DIII/IV as a single explanatory variable

We built LRM models [7] to investigate the influence of the dimerisation domain dissimilarity on the likelihood of interaction between transcriptional regulators. The linear regression mixture model with a single explanatory variable is written as follows:

(5) $$ X_{ij}|\left\{i\in C_{q},j\in C_{\ell}\right\} \sim\mathcal{N}\left(\mu_{\textit{q}\ell}+\beta_{\textit{q}\ell} Y_{ij},\sigma^{2}\right),   $$

where *X* is the weighted adjacency matrix (response distance matrix) and *Y* the explanatory distance matrix representing primary sequence dissimilarities between DIII/IV. As for GM models, *μ*_*q*,*ℓ*_ is the mean likelihood of interaction between proteins of two clusters. The regression parameter *β*_*q*,*ℓ*_ quantifies the effect of DIII/IV sequence disimilarity on interaction likelihood and is defined for each pair of clusters (*q*,*ℓ*) [7].

Introducing an explanatory variable enables to reduce the number of clusters selected by the ICL criterion: four clusters for the LRM model instead of five clusters for the GM-A model (Tables 2 and 6). The single-explanatory-variable 4-cluster LRM model (Table 7) exhibits a biologically meaningful structure with three clusters $C1^{\text {ARF+}}_{\texttt {LRM-1}}$, $C2^{\text {ARF-}}_{\texttt {LRM-1}}$ and $C3^{\text {IAA}}_{\texttt {LRM-1}}$ enriched respectively in ARF+, ARF- and Aux/IAA and an “outlier” cluster $C4^{\text {Outlier}}_{\texttt {LRM-1}}$ composed of ARF- and Aux/IAA; see Additional file 1: Figure S7 for the clustered valued ajdacency matrix with proteins sorted by increasing within-cluster distances. This composition is very similar to the one obtained with the 4-cluster GM-A model (87 *%* of match) but a bit less to the one obtained with the 4-cluster BM model (76 *%* of match) (Table 5).

(6) $${} {\small{\begin{aligned} &\hspace{70pt} \!\!\!\!C1^{\text{ARF+}}_{\texttt{LRM-1}} \hspace{5pt} C2^{\text{ARF-}}_{\texttt{LRM-1}} \hspace{5pt} C3^{\text{IAA}}_{\texttt{LRM-1}} \hspace{5pt} C4^{\text{Outlier}}_{\texttt{LRM-1}}\\ &D_{\text{LRM-1}}(q,l) = \left(\begin{array}{cccc} 0.025 & \qquad0.015 & \quad0.029 & {\phantom{0}}\quad0.022{\phantom{0}}\\ 0.015 & \qquad0.014 & \quad0.016 & {\phantom{0}}\quad0.016{\phantom{0}}\\ 0.029 & \qquad0.016 & \quad0.034 & {\phantom{0}}\quad0.021{\phantom{0}}\\ 0.022 & \qquad0.016 & \quad0.021 & {\phantom{0}}\quad0.022{\phantom{0}} \end{array}\right)\\ \end{aligned}}}   $$

**Table 6:** ICL criterion values and corresponding posterior model probabilities for single- (LRM-1) and two-explanatory-variable (LRM-2) linear regression mixture models

	No. clusters	1	2	3	4	5	6
LRM-1	ICL	−570.028	−343.172	−277.012	−272.276	−282.175	−290.605
	post. proba.	0	0	0.009	0.991	0	0
LRM-2	ICL	−532.263	−334.018	−293.711	−295.069	−312.373	−354.551
	post. proba.	0	0	0.795	0.205	0	0

**Table 7:** Composition of the four clusters obtained using the single-explanatory-variable LRM model

$C1^{\text {ARF+}}_{\texttt {LRM-1}}$	**ARF5** (0.022), **ARF6**, **ARF7**, **ARF8**, **ARF19** (0.024), IAA31 (0.027), IAA7 (0.029), IAA13 (0.029)
$C2^{\text {ARF-}}_{\texttt {LRM-1}}$	ARF1 (0.012), ARF10, ARF16, IAA6, IAA11, ARF4 (0.013), ARF14, ARF18, ARF2 (0.015), ARF13, ARF12 (0.016)
$C3^{\text {IAA}}_{\texttt {LRM-1}}$	IAA10 (0.029), IAA15, IAA14 (0.03), IAA12 (0.031), IAA1, IAA2, IAA18, IAA27 (0.033), IAA17, IAA19, IAA28, IAA4 (0.035), IAA16, IAA34, IAA3, IAA5,
	IAA8, IAA9 (0.037)
$C4^{\text {Outlier}}_{\texttt {LRM-1}}$	IAA29 (0.018), ARF22, IAA33, ARF20, IAA26, IAA32, IAA20, IAA30, ARF9 (0.026)

The ARF activators are in bold. The distance *D*(*i, q*) between protein *i* and cluster *q* to which it is assigned is given for the most central, the most peripheral and some other proteins of interest for interpretation. See Additional file 1: Figure S11 for the distance plot

Considering the between-cluster distance matrix (Eq. 6) we observe an increase of the ARF+ enriched within-cluster distance, while the other clusters show within- and between-cluster distances similar to the ones in the GM-A model (Eq. 4). The estimated regression coefficients of the linear regression models are given in Eq. 7; see Additional file 1: Figure S8 for a graphical representation of the regressions.

(7) $${} {\fontsize{8}{8}{\begin{aligned} &\hspace{86pt} \!\!\!\!\!\!\!\! C1^{\text{ARF+}}_{\texttt{LRM-1}} \hspace{5pt} C2^{\text{ARF-}}_{\texttt{LRM-1}} \hspace{7pt} C3^{\text{IAA}}_{\texttt{LRM-1}} \hspace{5pt} C4^{\text{Outlier}}_{\texttt{LRM-1}}\\ &\hat{\beta}_{\text{III/IV, LRM}}(q,\ell) =\left(\begin{array}{cccc} {\phantom{0}}1.024 & \quad0.097 & \quad0.305 & \quad0.701\\ {\phantom{0}}0.097 & \quad-0.092 & \quad-0.057 & \quad0.119\\ {\phantom{0}}0.305 & \quad-0.057 & \quad-0.031 & \quad-0.014\\ {\phantom{0}}0.701 & \quad0.119 & \quad-0.014 & \quad-0.081 \end{array} \right)\\ \end{aligned}}}   $$

**Fig. 6:**
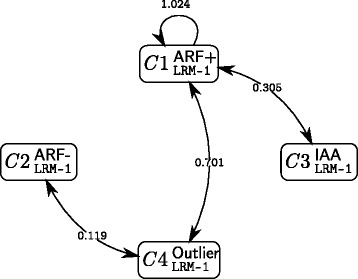
Influence of the dimerisation sequence distances on interaction likelihoods within the 4-cluster single-explanatory-variable LRM model. The estimated regression coefficients $\hat {\beta }_{\text {III/IV}}(q,\ell)$ are defined for each pair of clusters, but only those significantly different from zero are represented

We give in Fig. 6 a simplified representation of the influence of the dimerisation sequence distance on the likelihood of interaction between proteins of two clusters. We stress here that this representation cannot be compared with the connectivity graphs (Figs. 4 and 5) since they do not present the same information.

In the case of $C1^{\text {ARF+}}_{\texttt {LRM-1}}$, the estimated regression coefficients $\hat {\beta }_{\text {III/IV, LRM}}(q,\ell)$ (Eq. 7) show that the closer the dimerisation sequences, the less proteins in $C1^{\text {ARF+}}_{\texttt {LRM-1}}$ are likely to interact $\left (\hat {\beta }\left (C1^{\text {ARF+}}_{\texttt {LRM-1}},C1^{\text {ARF+}}_{\texttt {LRM-1}}\right)=1.024\right)$. However, as shown in Table 7, $C1^{\text {ARF+}}_{\texttt {LRM-1}}$ is not only made of ARF+ but also includes IAA31, 7 and 13. A closer look (Additional file 1: Figure S8, top-left panel) shows that the positive influence detected for within-$C1^{\text {ARF+}}_{\texttt {LRM-1}}$ interaction comes from the presence of the three Aux/IAA in this cluster. We observed mainly two separated groups (in addition to the homo-dimers): one with low interaction likelihoods (and low dimerisation sequence distances) that corresponds to ARF+ ⇔ARF+ and Aux/IAA ⇔Aux/IAA interactions, and another one with high interaction likelihoods (and higher dimerisation sequence distances), that corresponds to ARF+ ⇔Aux/IAA interactions (Additional file 1: Figure S8). This indicates that this result is most likely an artefact.

Considering the interaction between $C1^{\text {ARF+}}_{\texttt {LRM-1}}$ and $C3^{\text {IAA}}_{\texttt {LRM-1}}$, we also observed a weak but positive effect $\left (\hat {\beta }\left (C1^{\text {ARF+}}_{\texttt {LRM-1}},C3^{\text {IAA}}_{\texttt {LRM-1}}\right)\!=0.305\!\right)$ of dimerisation sequence distance on the interaction likelihood (the closer the sequences the less likely proteins interact). A closer inspection shows a less dispersed distribution of interaction likelihoods (Additional file 1: Figure S8), supporting the observed effect of dimerisation sequence distances on interaction likelihoods. Similar observations can be made for the interaction between $C1^{\text {ARF+}}_{\texttt {LRM-1}}$ and $C4^{\text {Outlier}}_{\texttt {LRM-1}} \left (\hat {\beta }\left (C1^{\text {ARF+}}_{\texttt {LRM-1}},C4^{\text {Outlier}}_{\texttt {LRM-1}}\right)=0.701\right)$. Surprisingly, no effect of dimerisation sequence distances on within-$C4^{\text {IAA}}_{\texttt {LRM-1}}$ interaction could be detected $\left (\hat {\beta }\left (C4^{\text {IAA}}_{\texttt {LRM-1}},C4^{\text {IAA}}_{\texttt {LRM-1}}\right)=-0.031\right)$. Apart from these observations, no other influence of dimerisation sequence distance on interaction likelihood could be identified using this model.

#### Linear regression mixture models with DIII and DIV as two explanatory variables

We next tested how each dimerisation sub-domain DIII and DIV could influence the interaction likelihood by incorporating in the LRM models two explanatory variables (one for each dimerisation sub-domain). The linear regression mixture model with two explanatory variables can be written as follows:

(8) $${} {\fontsize{8.3}{6}{\begin{aligned} X_{ij}|\!\left\{i\in C_{q},j\in C_{\ell}\right\} \sim\mathcal{N}\!\left(\mu_{\textit{q}\ell}+\beta_{\text{III},q\ell} Y_{\text{III},ij}+\beta_{\text{IV},q\ell} Y_{\text{IV},ij},\sigma^{2}\right), \end{aligned}}}   $$

The ICL criterion favours the 3-cluster two-explanatory-variable LRM model and with a non-significant difference (*Δ*ICL < 1.4) the 4-cluster two-explanatory-variable LRM model (Table 6). The cluster composition obtained with the 4-cluster two-explanatory-variable LRM model (Table 8) is very similar to the one obtained with the 4-cluster single-explanatory-variable LRM model (Table 7, 95 *%* of match) and with the GM-A model (Table 4, 91 *%* of match). The 4-cluster two-explanatory-variable LRM model has 3 clusters $C1^{\text {ARF+}}_{\texttt {LRM-2}}$, $C2^{\text {ARF-}}_{\texttt {LRM-2}}$ and $C3^{\text {IAA}}_{\texttt {LRM-2}}$ enriched respectively in ARF+, ARF- and Aux/IAA and an “outlier” cluster $C4^{\text {Outlier}}_{\texttt {LRM-2}}$; see Additional file 1: Figure S9 for the clustered valued adjacency matrix with proteins sorted by increasing within-cluster distances. The 4 clusters deduced from this LRM model have similar within- and between-cluster distances (Eq. 9) than the 4 clusters deduced from the single-explanatory-variable LRM model (Eq. 6).

(9) $${} {\small{\begin{aligned} &\hspace{73pt} \!\!\!\!C1^{\text{ARF+}}_{\texttt{LRM-2}} \hspace{5pt} C2^{\text{ARF-}}_{\texttt{LRM-2}} \hspace{5pt} C3^{\text{IAA}}_{\texttt{LRM-2}} \hspace{5pt} C4^{\text{Outlier}}_{\texttt{LRM-2}}\\ &D_{\text{LRM-2}}(q,l) =\left(\!\!\begin{array}{cccc} {\phantom{0}}0.025 & \hspace{5pt} \quad0.016 &\hspace{5pt} \quad0.029 &\hspace{5pt} \quad0.022{\phantom{0}}\\ {\phantom{0}}0.016 & \hspace{5pt} \quad0.014 &\hspace{5pt} \quad0.016 &\hspace{5pt} \quad0.017{\phantom{0}}\\ {\phantom{0}}0.029 & \hspace{5pt} \quad0.016 &\hspace{5pt} \quad0.034 &\hspace{5pt} \quad0.022{\phantom{0}}\\ {\phantom{0}}0.022 & \hspace{5pt} \quad0.017 &\hspace{5pt} \quad0.022 &\hspace{5pt} \quad0.023{\phantom{0}} \end{array}\right)\\ \end{aligned}}}   $$

**Table 8:** Composition of the four clusters obtained using the LRM model with two explanatory variables

$C1^{\text {ARF+}}_{\texttt {LRM-2}}$	**ARF5** (0.022), **ARF6**, **ARF7**, **ARF8**, **ARF19** (0.024), IAA31 (0.027), IAA7 (0.029), IAA13 (0.029)
$C2^{\text {ARF-}}_{\texttt {LRM-2}}$	ARF1 (0.012), ARF10, IAA6, IAA11, ARF4 (0.013), ARF14, ARF16, ARF18, IAA29, ARF20 (0.014), ARF12 (0.015), ARF13, ARF2 (0.016)
$C3^{\text {IAA}}_{\texttt {LRM-2}}$	IAA10 (0.029), IAA15, IAA14 (0.03), IAA12 (0.031), IAA1, IAA2, IAA18, IAA27 (0.033), IAA17, IAA19, IAA28, IAA4 (0.035), IAA16, IAA34, IAA3 (0.036), IAA5, IAA8, IAA9 (0.037)
$C4^{\text {Outlier}}_{\texttt {LRM-2}}$	IAA33 (0.018), ARF22, IAA30, ARF9, IAA20, IAA26, IAA32 (0.025)

The ARF activators are in bold.The distance *D*(*i, q*) between protein *i* and cluster *q* to which it is assigned is given for the most central, the most peripheral and some other proteins of interest for interpretation. See Additional file 1: Figure S12 for the distance plot

**Fig. 7:**
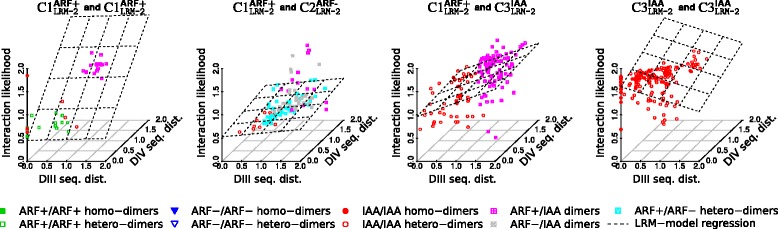
Example of estimated linear regressions based on a 4-cluster two-explanatory-variable LRM model. The regressions are for the pairs of clusters: $C1^{\text {ARF+}}_{\texttt {LRM-2}}$ and $C1^{\text {ARF+}}_{\texttt {LRM-2}}$, $C1^{\text {ARF+}}_{\texttt {LRM-2}}$ and $C2^{\text {ARF-}}_{\texttt {LRM-2}}$, $C1^{\text {ARF+}}_{\texttt {LRM-2}}$ and $C3^{\text {IAA}}_{\texttt {LRM-2}}$, $C3^{\text {IAA}}_{\texttt {LRM-2}}$ and $C3^{\text {IAA}}_{\texttt {LRM-2}}$. We highlighted the dimers types as indicated in the legend

The estimated regression coefficients for the two sub-domains are given in Eqs. 10 and 11; see Fig. 7 and Additional file 1: Figure S10 for graphical representations of the regressions.

(10) $${} {\fontsize{8}{8}{\begin{aligned} &\hspace{67pt} C1^{\text{ARF+}}_{\texttt{LRM-2}} \hspace{5pt} C2^{\text{ARF-}}_{\texttt{LRM-2}} \hspace{6pt} C3^{\text{IAA}}_{\texttt{LRM-2}} \hspace{7pt} C4^{\text{Outlier}}_{\texttt{LRM-2}}\\ &\hat{\beta}_{\text{III, LRM}}(q,\ell) =\left(\begin{array}{cccc} {\phantom{0}}0.021 & \hspace{3pt} \quad0.079 & \hspace{3pt} \quad0.294 & \quad0.569 \\ {\phantom{0}}0.079 & \hspace{3pt} \quad0.004 & \hspace{3pt} \quad0.194 & \quad0.088 \\ {\phantom{0}}0.294 & \hspace{3pt} \quad0.194 & \hspace{3pt} \quad-0.268 & \quad-0.219 \\ {\phantom{0}}0.569 & \hspace{3pt} \quad0.088 & \hspace{3pt} \quad-0.219 & \quad-0.050 \\ \end{array}\right)\\ \end{aligned}}}   $$

(11) $${} {\fontsize{8}{8}{\begin{aligned} &\hspace{67pt} C1^{\text{ARF+}}_{\texttt{LRM-2}} \hspace{5pt} C2^{\text{ARF-}}_{\texttt{LRM-2}} \hspace{6pt} C3^{\text{IAA}}_{\texttt{LRM-2}} \hspace{5pt} C4^{\text{Outlier}}_{\texttt{LRM-2}}\\ &\hat{\beta}_{\text{IV, LRM}}(q,\ell) =\left(\begin{array}{cccc} {\phantom{0}}0.887 & \quad0.109 & \quad0.052 & \quad0.069{\phantom{0}}\\ {\phantom{0}}0.109 & \quad-0.045 & \quad-0.138 & \quad0.037{\phantom{0}}\\ {\phantom{0}}0.052 & \quad-0.138 & \quad0.297 & \quad0.208{\phantom{0}}\\ {\phantom{0}}0.069 & \quad0.037 & \quad0.208 & \quad0.004{\phantom{0}}\\ \end{array}\right)\\ \end{aligned}}}   $$

**Fig. 8:**
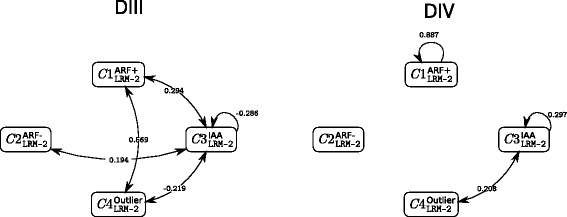
Influence of the dimerisation sequence distances on interaction likelihoods within the 4-cluster two-explanatory-variable LRM model. The estimated DIII and DIV regression coefficients $\hat {\beta }_{\text {III}}(q,\ell)$ and $\hat {\beta }_{\text {IV}}(q,\ell)$ are defined for each pair of clusters, but only those significantly different from zero are represented

We give in Fig. 8 two representations of the influence of dimerisation sub-domain sequence distance on interaction likelihood and thus on network topology.

For $C1^{\text {ARF+}}_{\texttt {LRM-2}}$ within-cluster interactions, the closer the DIV sequence distances the higher the interaction likelihood while DIII sequence distances had no effect on these within-cluster interactions. However, given that the composition of $C1^{\text {ARF+}}_{\texttt {LRM-2}}$ was identical in the 4-cluster LRM model with a single or two explanatory variables, these results are likely artefactual and linked to the fact that the ARF+ enriched cluster contains three Aux/IAA that contribute to the detected dimerisation sequence influence.

The $C3^{\text {IAA}}_{\texttt {LRM-2}}$ within-cluster interactions presents an opposite behaviour when analyzing the influence of the two dimerisation sub-domains: the closer the DIII sequences, the higher the interaction likelihood $\left (\hat {\beta }_{\text {III}}\left (C3^{\text {IAA}}_{\texttt {LRM-2}},C3^{\text {IAA}}_{\texttt {LRM-2}}\right)=-0.268\right)$; the farther the DIV sequences, the higher the interaction likelihood $\left (\hat {\beta }_{\text {IV}}\left (C3^{\text {IAA}}_{\texttt {LRM-2}},C3^{\text {IAA}}_{\texttt {LRM-2}}\right)=0.297\right)$. Counteracting effects of the same order of magnitude for the two sub-domains thus likely explain why we could not observe any influence of dimerisation sequence distances on interaction likelihood with the single-explanatory-variable LRM model $\left (\hat {\beta }\left (C3^{\text {IAA}}_{\texttt {LRM-1}},C3^{\text {IAA}}_{\texttt {LRM-1}}\right)=-0.031\right)$.

Domain-specific effects were also found for the interaction between $C1^{\text {ARF+}}_{\texttt {LRM-2}}$ and $C3^{\text {IAA}}_{\texttt {LRM-2}}$. DIII sequence distance is positively related to interaction likelihood $\left (\hat {\beta }_{\text {III}}\left (C1^{\text {ARF+}}_{\texttt {LRM-2}},C3^{\text {IAA}}_{\texttt {LRM-2}}\right)=0.294\right)$, while no –or a very limited– effect of DIV sequence distances is observed $\left (\hat {\beta }_{\text {IV}}\left (C1^{\text {ARF+}}_{\texttt {LRM-2}},C3^{\text {IAA}}_{\texttt {LRM-2}}\right)=0.052\right)$. This is in agreement with the effect $\left (\hat {\beta }\left (C1^{\text {ARF+}}_{\texttt {LRM-1}},C3^{\text {IAA}}_{\texttt {LRM-1}}\right)=0.305\right)$ detected within the single-explanatory-variable LRM model and indicates that the effect of dimerisation sequence distance on interaction likelihood is mostly linked to DIII, with little or no contribution of DIV.

Finally concerning interactions between $C2^{\text {ARF-}}_{\texttt {LRM-2}}$ and $C3^{\text {IAA}}_{\texttt {LRM-2}}$, a weak opposite effect was detected with the two-explanatory-variable LRM model for each domain $\left (\hat {\beta }_{\text {III}}\left (C2^{\text {ARF-}}_{\texttt {LRM-2}},C3^{\text {IAA}}_{\texttt {LRM-2}}\right)=0.194\right)$ and $\left.\hat {\beta }_{\text {IV}}\left (C2^{\text {ARF-}}_{\texttt {LRM-2}},C3^{\text {IAA}}_{\texttt {LRM-2}}\right)=-0.138 \right)$. Again this could not be detected with the single-explanatory-variable LRM model $\left (\hat {\beta }\left (C2^{\text {ARF-}}_{\texttt {LRM-1}},C3^{\text {IAA}}_{\texttt {LRM-1}}\right)=-0.057\right)$, most likely because of opposite contributions from the two sub-domains.

## Conclusions

### Interpretation of the auxin signalling network clustering and of the contribution of domain III/IV primary sequences

Our clustering analysis provides interesting insight on the underlying biology. First and in accordance with previous work [4], the different models strongly support the idea that the auxin signalling network can be simplified in three biologically meaningful groups, corresponding roughly to the ARF+, ARF- and Aux/IAA (but with an additional outlier group, see below) and showing specific interaction behaviours. The strong interaction likelihood between ARF+ and Aux/IAA was expected from the putative molecular model reviewed in [2]. This suggests that most of the Aux/IAA repress transcriptional activity of ARF+ when a low concentration of auxin is encountered. However, the weak likelihood of interaction between ARF- and Aux/IAA, and between ARF- and ARF+ remains a surprising conclusion (that was highlighted by [4]), given the overall good conservation of DIII/IV in ARF- proteins. Further experiments and analyses need to be conducted to unveil the role of DIII/IV in ARF- and its possible contribution to the auxin signalling pathway.

Using LRM models to investigate the influence of protein sequence distances on the auxin signalling network is a first attempt to establish a direct link between protein primary sequences and interaction network topology. By first using a single-explanatory-variable LRM model, we uncovered a rather counter-intuitive contribution of the primary sequence for a few between-cluster interactions. Notably proteins from the ARF+ enriched cluster interact more likely with proteins from the Aux/IAA enriched cluster that have more distant dimerisation sequences. This suggests that the likelihood of interaction between ARF+ and Aux/IAA increases with the evolutionary distance between DIII/IV sequences. A similar observation could be made for the ARF+ enriched cluster and the outlier cluster, further suggesting that facilitated interactions between more distant proteins could contribute significantly to the structuring of the auxin signalling network. Concerning the ARF+ enriched within-cluster interactions, we detected a positive relationship between protein distance and interaction likelihood. However, this is likely an artefact due to the presence of three Aux/IAA in this cluster, preventing us from drawing conclusion from this observation.

The two-explanatory-variable LRM models yielded a more precise view by identifying sub-domain specific effects. Our results show that DIII explains most of the effect of DIII/IV sequence on the likelihood of interaction between ARF+ and Aux/IAA. Recent structural analyses of DIII/IV [12–15] showed that DIII and DIV mediate interactions between ARF+ and Aux/IAA through two charged interfaces: one face mostly positive and one face mostly negative. DIII contributes principally to the positive face, while DIV contributes to the negative face of these interaction domains. This structure allows for bi-directional interactions. Finding that changes in the primary sequence of DIII alone influence ARF+ ⇔Aux/IAA interaction likelihood then suggests that changes on a single face of the protein impact the global interaction capability. Analysing the contribution of each sub-domain also highlighted several antagonistic influences, thus explaining why no effect was detected with the single-explanatory-variable LRM model in some cases. This suggests that changes within the primary sequence of one sub-domain that could influence the interaction likelihood, can be counteracted by changes within the primary sequence of the other sub-domain, an effect that could have occurred during evolution of DIII/IV.

So far DIII/IV structures have been obtained for 4 transcriptional regulators of auxin [12–15]. Obtaining further protein structures, although challenging, could allow testing the hypotheses emerging from our clustering approach. Other strategies would also allow testing further the link between interaction likelihood and protein dissimilarities:

- creating a library of mutated version of DIII and DIV for each element of the network to artificially enlarge the network size;
- generating similar Y2H data for other species, such as rice or tomato, which also possess large families of auxin-related transcriptional regulators.

This would be particularly useful for small clusters such as the ARF+ enriched cluster, for which the regression model is constrained by the rather limited number of transcriptional regulators. However, it is important to stress here that the Y2H experiment was performed using additional sequences than DIII/IV for Aux/IAA (full length protein were used: [4]). Although there is no evidence that other Aux/IAA domains contribute to binding, we cannot eliminate the possibility that this introduces a bias that could affect the analysis of the influence of DIII/IV primary sequence distance on interaction likelihood. Testing the interaction capacity using only DIII/IV protein sequences for both Aux/IAA and ARF could be useful in the future to address this question. Note also that [16] has suggested an effect of the ARF middle region on interactions between Aux/IAA and ARF, thus implying that the interaction landscape could be more complex than the one established in our analysis.

It is finally interesting to compare the composition of the outlier cluster obtained with the different models (BM, GM, single- and two-explanatory-variable LRM models). Four proteins (ARF22, IAA26, 32 and 33) were systematically assigned to the outlier cluster while three others (ARF9, IAA20 and 30) were assigned to the outlier cluster for all models estimated on the basis of the valued graph (GM, single- and two-explanatory-variable LRM models). The composition of the outlier cluster is thus largely conserved for the different models. While this could be interpreted as a consequence of noise in the Y2H experiments affecting more specifically these proteins, analysis of the distribution of interaction likelihood involving this cluster suggest that proteins in this cluster might actually have a peculiar behaviour in the network (Additional file 1: Figure S9). The outlier cluster is characterized by an highly dispersed interaction likelihood. Proteins identified in the outlier cluster could thus be involved in specific interactions within the network, possibly highlighting an unsuspected function for these proteins in the regulation of auxin signalling.

## Methods

### Testing the Aux/IAA - ARF interaction capability

The Y2H experiment is a bio-engineered tool based on the Gal-4 transcription factor from yeast *Saccharomyces Cerevisiae*. The Gal-4 transcription factor is made of an N-terminal DNA binding domain (BD) and a C-terminal activation domain (AD). These two sub-parts have been artificially separated, and tagging each with proteins allow to test for their interaction capability.

#### Yeast-2-hybrid protein interaction testing

In the original screening presented in [4], we manage to test the interaction capability of all members of the Aux/IAA - ARF family, except for ARF 15, 21 and 23. The interaction screening was therefore conducted on 49 transcriptional regulators, representing 1225 tested interactions. In order to be thorough, each interaction was tested both ways, meaning each protein was append to both AD and BD in two separate repetitions (e.g. AD-ARF1 v.s. BD-ARF2 and AD-ARF2 v.s. BD-ARF1). Finally, considering that this screening method can present false positives, two independent biological tests were conducted for each way. Overall, this represents a total of 4900 test results to analyze.

In this paper, we aim at modelling the influence of dissimilarities between dimerisation sequences on transcriptional regulator interactions. We thus had to remove the members of the Aux/IAA - ARF familly that does not possess the protein-protein dimerisation domain, namely ARF3 and 17. This brings the number of proteins implicated in the network down to 47. Finally, ARF11 does not present any connexion in the binary network. In order to ease the comparison of the random graph clustering model outputs we chose to remove ARF11 from the analyses.

#### Reporting genes

The *β*-galactosidase (*β*-gal) is an enzyme hydrolyzing X-Gal (or 5-bromo-4-chloro-3-indolyl-beta-D-galactopyranosid) into a blue compound revealing its activity (i.e the interaction between proteins). The other reporting gene encodes a protein called imidazoleglycerol-phosphate dehydratase (HIS3) which catalyses the sixth step in histidine biosynthesis. It is also from *S. Cerevisiae* and allow the yeast to produce histidine and thus to survive in an histidine-free medium.

#### Data description

The X-Gal test is based on a blue coloration of the media where yeasts are developing. The ordered marks for the X-Gal test were ‘-’, ‘-?’, ‘?’, ‘+?’, ‘+’, ‘++’, ‘+++’. We chose to use this full ordinal scale for computing standardized distances in order to build valued graphs. The four first marks ‘-’, ‘-?’, ‘?’, ‘+?’, were not distinguished and assimilated to ‘-’ in [4] for defining a threshold for the X-Gal test. We fixed this threshold between ‘+?’ and ‘+’ (in the original ordinal scale) as in [4] in order to build binary graphs.

The HIS3 test is based on the capability of yeasts to synthesize histidine in an histidine-free medium. It can be viewed as an estimation of histidine synthesis capability upon function recovery. To assess for this synthesis capacity, a ratio of optical densities (ODs) between yeast growth in a medium without histidine and with histidine was used: {OD histidine-free medium}/ {OD histidine-rich medium}. For detailed explanations on the test outputs used in the Y2H screen, see [4].

### Network binarisation

#### Mixture model for optical density ratios

We estimated a three-component Gaussian mixture model $ \sum _{i=1}^{3} \alpha _{i} f_{i}\left (z;\mu _{i}, {\sigma _{i}^{2}}\right) $ on the basis of the overall OD ratio sample (HIS3 test) using the mclust R package [17]. The three components were selected using the Bayesian information criterion (BIC). We then investigated possible consistencies between limits between components (given by the values where the posterior probabilities of successive components are equal) and limits between successive marks for the X-Gal test. The first two components correspond to almost only X-Gal marks < ‘+’ while the last one corresponds mostly to marks ≥ ‘+’; see Fig. 2. The threshold for the HIS3 test was then fixed close to the limit between the second and the third component and the threshold for the X-Gal test between marks < ‘+’ and ≥ ‘+’.

#### Decision rules

Because it is a two-way two-reporting-gene experiment, there are several possible test configurations which define the presence or absence of interaction for each tested interaction. In the following tables we give configurations potentially reflecting the ‘presence of interaction’, where we define a given test as “positive” (+) or “negative” (-) when its result is respectively above or below the defined thresholds: Configuration 1: all the tests are positive,



Configuration 2: only one test is not positive,



Configuration 3: only one way is positive for both reporter genes,



Configuration 4: one reporter gene is positive in each way,



Configuration 5: only one reporter gene is positive both ways,



An analysis -not detailed here- allowed us to state that the fifth configuration (only one reporter gene is positive both ways) is unreliable. We therefore discarded this case when defining the presence or absence of interaction for the binary network.

### Dimerisation domain primary sequences

The protein sequences were obtained using the accession numbers of Aux/IAA and ARF presenting a dimerisation domain (ARF 3, 17 and 23 were thus excluded); see availability of supporting data for list of AGIs. Sub-sequences corresponding to DIII and DIV were obtained by first making a multiple alignment of the whole protein sequences using Clustal-W [10]. Then, we searched for highly conserved regions using Gblocks 0.91b [11]; see availability of supporting data for list of used parameters. We subsequently found three conserved regions, the last two corresponding to DIII and DIV; for more information, see Additional file 1: Figure S6.

The flanking positions detected for domains III and IV from the full amino acid sequences were respectively [1275-1307] and [1344-1376]. Both conserved domains have a length of 32 amino acids. The sequence for DIII/IV is obtained from the concatenation of the two separate domains. We also conducted an analysis with slightly extended flanking positions [1272-1307] and [1344-1376], but this did not lead to significant changes in the analyses.

### Linear regression mixture models for valued random graphs

The first version of the stochastic block model (SBM) was introduced in [18] and assumes that vertices are distributed into clusters and that the probability for an edge to exist between two vertices depends on the clusters the two vertices belong to, as described in Eq. (1). The LRM model used here is an extension to valued graphs with explanatory variables of the model introduced in [18]. An estimation method based on an expectation-maximization (EM) algorithm, with a variational approximation in the E-step was proposed in [7]. We briefly remind here some key ingredients of the estimation procedure. An implementation of the algorithm used in this study is provided by wmixnet [19].

#### Definition of linear regression mixture models

We consider a graph with *n* vertices (*i*=1,…*n*). Each vertex is assumed to belong to an (unobserved) cluster *C*_*q*_ among *Q* possible clusters *C*_1_,…*C*_*Q*_. The probability for a given vertex to belong to cluster *q* is denoted by $\alpha _{q} \left (\sum _{q=1}^{Q} \alpha _{q} = 1\right)$. The vertex memberships are supposed to be independent. For each pair of vertices (*i, j*), *X*_*ij*_ denote the weight of the edge between them and **Y**_*ij*_ the vector of explanatory variables associated with this pair of vertices. In the proposed model, the edge weights are independent conditionally on the vertex membership:

$$X_{ij} | \{i \in C_{q}, j \in C_{\ell}\} \sim \mathcal{N}\left(\mu_{\textit{q}\ell} + \textbf{Y}_{ij}^{\intercal} \textbf{b}_{\textit{q}\ell},\sigma^{2}\right). $$

All the models considered here (except Model (1)) can be casted in this framework, taking for Model (2): **Y**_*ij*_=*∅*, **b**_*qℓ*_=*∅* ; for Model (5): $\textbf {Y}_{\textit {ij}}^{\intercal } =\, [\!Y_{\textit {ij}}]$, $\textbf {b}_{\textit {q}\ell }^{\intercal } =\, [\!\beta _{\textit {q}\ell }]$; and for Model (8): $\textbf {Y}_{\textit {ij}}^{\intercal } = \left [Y_{\text {III}, ij} \; Y_{\text {IV}, ij}\right ]$, $\textbf {b}_{\textit {q}\ell }^{\intercal } = \left [\beta _{\text {III}, q\ell } \; \beta _{\text {IV}, q\ell }\right ]$.

Note that all these models are heterogeneous versions of the regression models considered in [7], since both the constants *μ* and the regression coefficients *β* depend on the vertex membership. As a consequence, such a model with *d* explanatory variables and *Q* clusters involves (*Q*−1) independent membership probabilities *α*_*q*_, *Q*^2^ constants *μ*_*qℓ*_ and *dQ*^2^ regression coefficients *β*_*qℓ*_, that is (*Q*−1)+*Q*^2^(*d*+1) independent parameters.

#### Statistical methods for linear regression mixture models

The estimation of parameters, and the prediction of the vertex membership is made by a variational EM algorithm, first introduced for SBM in [6]. This algorithm is similar to a standard EM algorithm [20], since it alternates until convergence the determination of the conditional distribution of the vertex membership given the observed data (E-step) and the estimation of the parameters (M-step). The estimation formulas used in the M-step are given in [7].

In the case of SBM, the E-step cannot be calculated in an exact manner, as it would require to enumerate all possible vertex memberships, which is not possible even for a moderate network size. A variational approximation is used to circumvent this problem. Let *τ*_*iq*_ be the conditional probability for vertex *i* to belong to cluster *q* given the observed edge weights. An approximation of *τ*_*iq*_ is computed using the following fixed-point formula:

$${} {\small{\begin{aligned} \tau_{iq}\! \propto \alpha_{q} \prod_{j \neq i} \prod_{\ell}\! \left(\phi_{ij}^{q\ell} \right)^{\tau_{\textit{j}\ell}}\!, \,\, \text{where} \ \ \phi_{ij}^{q\ell} = \phi\left(\!\frac{X_{ij} - \mu_{\textit{q}\ell} - \textbf{Y}_{ij}^{\intercal} \textbf{b}_{\textit{q}\ell}}{\sigma}\! \right)\!, \end{aligned}}} $$

where *ϕ* stands for the probability density function of the standard Gaussian distribution. Each step of the variational EM algorithm can be shown to increase a lower bound $\mathcal {J}$ of the log-likelihood of the observed data which can be rewritten as:

$${} \mathcal{J} = \sum_{i, q} \tau_{iq} \log \alpha_{q} + \sum_{i, j, q, \ell} \tau_{iq} \tau_{\textit{j}\ell} \log \left(\phi_{ij}^{q\ell}\right) - \sum_{i, q} \tau_{iq} \log \tau_{iq}. $$

A model selection criterion is needed to choose the number of clusters. To this aim, we apply the ICL criterion derived in [6]. This criterion relies on a double penalty: one for the membership probabilities *α*_*q*_ that are associated with the *n* vertices and one for the regression parameters (*μ*_*qℓ*_,*β*_*qℓ*_) that are associated with the *n*(*n*−1)/2 edges. It finally writes as:

$$ICL = \mathcal{J} - \frac{Q-1}2 \log n - \frac{Q^{2}(d+1)}2 \log \frac{n(n-1)}2. $$

### Response distance matrix: standardized distances between transcriptional regulators

The Y2H analysis involves two independent tests, the X-Gal and the HIS3 tests. The output of the X-Gal test can be interpreted as a distance defined on an ordinal scale (from no interaction to strong interaction) while the output of the HIS3 test can be interpreted as a distance defined on a ratio scale (between 0 and 1.7). Combining these observed distances requires a standardization procedure. The objective of standardization is to avoid dependency on the elementary distance type and scale. In the case of an ordinal distance (X-Gal test), observed distances are replaced by ranked distances

$$\text{Rank}(y_{ij})=\frac{1}{2}+ \sum\limits_{n=0}^{y_{ij}-1}f_{n}+\frac{f_{y_{ij}}}{2}, $$

where *y*_*ij*_ is the output of the X-Gal test for proteins *i* and *j*, and *f*_*n*_ is the frequency of mark *n* (the possible marks are assumed to be represented as contiguous positive integers). In this case, the normalization quantity is the mean rank (1+*N*^2^)/2, where *N* is the number of proteins.

The ratio-scaled distance (HIS3 test) can be either treated as an interval-scaled distance or as an ordinal distance. Considering that the response curve of the HIS3 test is monotone but highly non-linear and is close to a Michaelis–Menten kinetics, we chose to consider the output of the HIS3 test as a distance defined on an ordinal scale for standardization. Observed distances are replaced by the ranked distances Rank(*y*_*ij*_) for the X-Gal test and Rank(*z*_*ij*_) for the HIS3 test, and the standardized distances are:

$$\begin{aligned} x_{ij} &=w_{\text{X-Gal}}\frac{\text{Rank}(y_{ij})+\text{Rank}(y_{ji})}{1+N^{2}}\\ &\quad+w_{\text{HIS3}}\frac{\text{Rank}(z_{ij}) +\text{Rank}(z_{ji})}{1+N^{2}}, \end{aligned} $$

where *w*_X-Gal_ and *w*_HIS3_ are the weights of the X-Gal and HIS3 tests with *w*_X-Gal_+*w*_HIS3_=1. It should be noted that a single marginal distribution was considered for each test used in the two possible configurations (bait or prey) in order to standardize the distances. In the case of missing test values, the distances can be straightforwardly adapted. If *z*_*ji*_ is missing, we obtain:

$${} x_{ij} =w_{\text{X-Gal}}\frac{\text{Rank}(y_{ij})+\text{Rank}(y_{ji}) } {1+M_{\text{X-Gal}}}+w_{\text{HIS3}}\frac{\text{Rank}(z_{ij}) } {(1+M_{\text{HIS3}})/2}, $$

where *M*_X-Gal_ is the number of X-Gal test values, and *M*_HIS3_ is the number of HIS3 test values.

The distance matrices {*x*_*ij*_;*i,j*=1,…,*N*} corresponding to (*w*_X-Gal_,*w*_HIS3_)=(1,0), (0.75,0.25), (0.5,0.5), (0.25,0.75), (0,1) were built and tested.

### Distances between dimerisation domain primary sequences

To use the primary sequence information as an explanatory variable in LRM models, we have to define a distance between two protein sequences. PROTDIST allows to compute such distances by using amino acid substitution models. One can choose between five different models, and we tested three of them: PAM, JTT and PMB. PMB which performed poorly was not used in the analyses. Finally, PAM and JTT outputs being rather similar, we focused on the PAM model, since it seems to be the most common one to date. For more information about the protein substitution models, see the PROTDIST documentation (http://evolution.genetics.washington.edu/phylip/doc/protdist.html).

### Assessing the quality of the clustering

We assessed the quality of the clustering obtained by evaluating the separability of the clusters and the dispersion of the proteins within the clusters. Since, in our case, the assignment of proteins to clusters is almost deterministic (i.e. *τ*_*iq*_≃1 for a unique cluster *q* and *τ*_*iℓ*_≃0 for *ℓ*≠*q* where *τ*_*iq*_ is the posterior probability of assigning protein *i* to cluster *q*), this assignment can be viewed as a partition. The model parameters, which parametrized the edges of the graph, cannot be used directly to define dispersion measures of the proteins assigned to a given cluster. We thus used the adjacency information to derive dissimilarity measures for the proteins. The distance $D(i,j)=\sum _{k}|x_{\textit {ik}}-x_{\textit {jk}}|/N$ between the *i*th and *j*th rows of the weighted adjacency matrix {*x*_*ij*_;*i,j*=1,…,*N*} quantifies the difference in connectivity profile between proteins *i* and *j*. In the case of the binary adjacency matrix, this distance is the Sokal-Michener distance between proteins *i* and *j* [21]: $D(i,j)=\sum _{k} I(x_{\textit {ik}}\neq x_{\textit {jk}})/N$, where *I*() denotes the indicator function. This is the proportion of mismatches between the *i*th and *j*th rows of the adjacency matrix.

The distance between protein *i* and cluster *q* is given by:

$$D(\textit{i,q}) = \frac{{\sum_{j\neq i}}\tau_{jq} {\sum_{k}} |x_{ik}-x_{jk}|} {\left\{{\sum_{j\neq i}}\tau_{jq}\right\} N}. $$

If the proteins are deterministically assigned to a given cluster, this distance simplifies to

$$\begin{array}{*{20}l} D(i,q) & =\frac{{\sum_{j\in q;j\neq i}}\sum_{k} |x_{ik}-x_{jk}|}{(n_{q}-1)N}\qquad i\in q,\\ D(i,q) & =\frac{{\sum_{j\in q}}\sum_{k} |x_{ik}-x_{jk}|}{n_{q}N}\qquad\quad\;\: i\notin q, \end{array} $$

where *n*_*q*_ is the number of proteins assigned to cluster *q*.The distance between cluster *q* and cluster *ℓ* can be directly derived as

$$\begin{array}{*{20}l} D(q,q) & =\frac{\sum_{i,j\in q;i\neq j}\sum_{k} |x_{ik}-x_{jk}|}{n_{q}(n_{q}-1)N},\\ D(q,\ell) & =\frac{\sum_{i\in q}\sum_{j\in \ell}\sum_{k} |x_{ik}-x_{jk}|}{n_{q}n_{\ell}N} \qquad q\neq \ell. \end{array} $$

The within- and between-cluster distances can then be defined as

$${} {\small{\begin{aligned} D_{\text{within}}(q) & = D(q,q)\qquad\qquad\qquad\qquad\qquad\;\:\text{within cluster,}\\ D_{\text{between}}(q) & =\frac{\sum_{i\in q}\sum_{j\notin q}\sum_{k} |x_{ik}-x_{jk}|}{n_{q}(N-n_{q})N}\qquad\text{between cluster.} \end{aligned}}} $$

### Availability of supporting data

#### Original Yeast-2-Hybrid data for Aux/IAA - ARF interaction tests

All Y2H interaction results for X-Gal and HIS3 reporters are available in supplementary data of [4].

#### Aux/IAA - ARF protein sequences

Protein sequences can be found within *Arabidopsis thaliana* proteins banks such as Swiss-Prot Protein Database http://www.expasy.org/ using the following 52 accession numbers: [Swiss-Prot:Q8L7G0, Swiss-Prot:Q94JM3, Swiss-Prot:O23661, Swiss-Prot: Q9ZTX9, Swiss-Prot:P93024, Swiss-Prot:Q9ZTX8, Swiss-Prot:P93022, Swiss-Prot:Q9FGV1, Swiss-Prot: Q9XED8, Swiss-Prot:Q9SKN5, Swiss-Prot:Q9ZPY6, Swiss-Prot:Q9XID4, Swiss-Prot:Q9FX25, Swiss-Prot: Q9LQE8, Swiss-Prot:Q9LQE3, Swiss-Prot:Q93YR9, Swiss-Prot:Q84WU6, Swiss-Prot:Q9C5W9, Swiss-Prot: Q8RYC8, Swiss-Prot:Q9C7I9, Swiss-Prot:Q9C8N9, Swiss-Prot:Q9C8N7, Swiss-Prot:Q9LP07, Swiss-Prot: Q38828, Swiss-Prot:Q38829, Swiss-Prot:Q38830, Swiss-Prot:Q38831, Swiss-Prot:Q38832, Swiss-Prot: Q9C966, Swiss-Prot:O24407, Swiss-Prot:P93830, Swiss-Prot:O24408, Swiss-Prot:O24409, Swiss-Prot:P49677, Swiss-Prot:O24410, Swiss-Prot:Q8LAL2, Swiss-Prot:Q9ZSY8, Swiss-Prot:Q9XFM0, Swiss-Prot:Q93WC4, Swiss-Prot:P49678, Swiss-Prot:Q9M1R4, Swiss-Prot: Q8H174, Swiss-Prot:Q8RYC6, Swiss-Prot:Q9FKM7, Swiss-Prot:Q9C5X0, Swiss-Prot:Q38822, Swiss-Prot:P33077, Swiss-Prot:P33078, Swiss-Prot:Q38824, Swiss-Prot:Q38825, Swiss-Prot:Q38826, Swiss-Prot:Q38827].

#### Domains III and IV sub-sequences

To obtain protein sub-sequences corresponding to conserved domains III and IV, we used the following parameters in Gblocks:

- Minimum Number Of Sequences For A Conserved Position: 25
- Minimum Number Of Sequences For A Flanking Position: 25
- Maximum Number Of Contiguous Nonconserved Positions: 8
- Minimum Length Of A Block: 10
- Allowed Gap Positions: With Half
- Use Similarity Matrices: Yes

See Additional file 1: Figure S6 for a detailed view of the aligned sequences and the conserved sub-sequences.

## Additional files

Additional file 1
**Supplemental Figures.** This file contains the following: histograms of optical density ratios (HIS3 test) for the successive X-Gal marks in **Figure S1.** The adjacency matrix obtained using 3 HIS3 thresholds in **Figure S2.** The adjacency matrices for the 4-cluster and 6-cluster BM models in **Figure S3.** The ranked average distances between transcriptional regulators for the 4-cluster GM-A model in **Figure S4.** The valued adjacency matrix for the 4-cluster GM-A model in **Figure S5.** The multiple alignment of amino acid sequences of the transcriptional regulators in **Figure S6.** The valued adjacency matrix for the 4-cluster single-explanatory-variable LRM model in **Figure S7.** The linear regressions for each pair of clusters within the 4-cluster single-explanatory-variable LRM model in **Figure S8.** The valued adjacency matrix for the 4-cluster two-explanatory-variable LRM model in **Figure S9.** The linear regressions for each pair of clusters within the 4-cluster two-explanatory-variable LRM model in **Figure S10.** the ranked average distances between transcriptional regulators for the 4-cluster single-explanatory-variable LRM model in **Figure S11.** and the ranked average distances between transcriptional regulators for the 4-cluster two-explanatory-variable LRM model in **Figure S12.** (PDF 1894 kb)

Additional file 2
**Supplemental Tables.** This file contains between-cluster distance matrices for the 6-cluster BM model in **Table S1.** and the 5-cluster GM-A model in **Table S2.** (PDF 75.2 kb)

## Abbreviations

Aux/IAA: AUXIN/INDOLE-3-ACETIC ACID
ARF: AUXIN RESPONSE FACTOR
CTD: C-terminal dimerisation domain
DBD: DNA binding domain
DIII/IV: Domain III/IV
DIII: Domain III
DIV: Domain IV
Y2H: Yeast-2-hybrid
AD: Activation domain
BD: Binding domain
GM: Gaussian mixture
BM: Bernoulli mixture
ICL: Integrated completed likelihood
LRM: Linear regression mixture
OD: Optical density
BIC: Bayesian information criterion
SBM: Stochastic block model
EM: Expectation-maximization
