# Supplementary material for: Modelling the influence of dimerisation sequence dissimilarities on the auxin signalling network
Source: BMC Syst Biol. 2016 Mar 1;10:22. doi: 10.1186/s12918-016-0254-7 (PMC4774195; doi:10.1186/s12918-016-0254-7)
Supplement: Additional file 1 — Supplemental Figures. This file contains the following: histograms of optical density ratios (HIS3 test) for the successive X-Gal marks in Figure S1. The adjacency matrix obtained using 3 HIS3 thresholds in Figure S2. The adjacency matrices for the 4-cluster and 6-cluster BM models in Figure S3. The ranked average distances between transcriptional regulators for the 4-cluster GM-A model in Figure S4. The valued adjacency matrix for the 4-cluster GM-A model in Figure S5. The multiple alignment of amino acid sequences of the transcriptional regulators in Figure S6. The valued adjacency matrix for the 4-cluster single-explanatory-variable LRM model in Figure S7. The linear regressions for each pair of clusters within the 4-cluster single-explanatory-variable LRM model in Figure S8. The valued adjacency matrix for the 4-cluster two-explanatory-variable LRM model in Figure S9. The linear regressions for each pair of clusters within the 4-cluster two-explanatory-variable LRM model in Figure S10. the ranked average distances between transcriptional regulators for the 4-cluster single-explanatory-variable LRM model in Figure S11. and the ranked average distances between transcriptional regulators for the 4-cluster two-explanatory-variable LRM model in Figure S12. (PDF 1894 kb) [file 12918_2016_254_MOESM1_ESM.pdf]

Additional file 1 — Supplemental Figures.

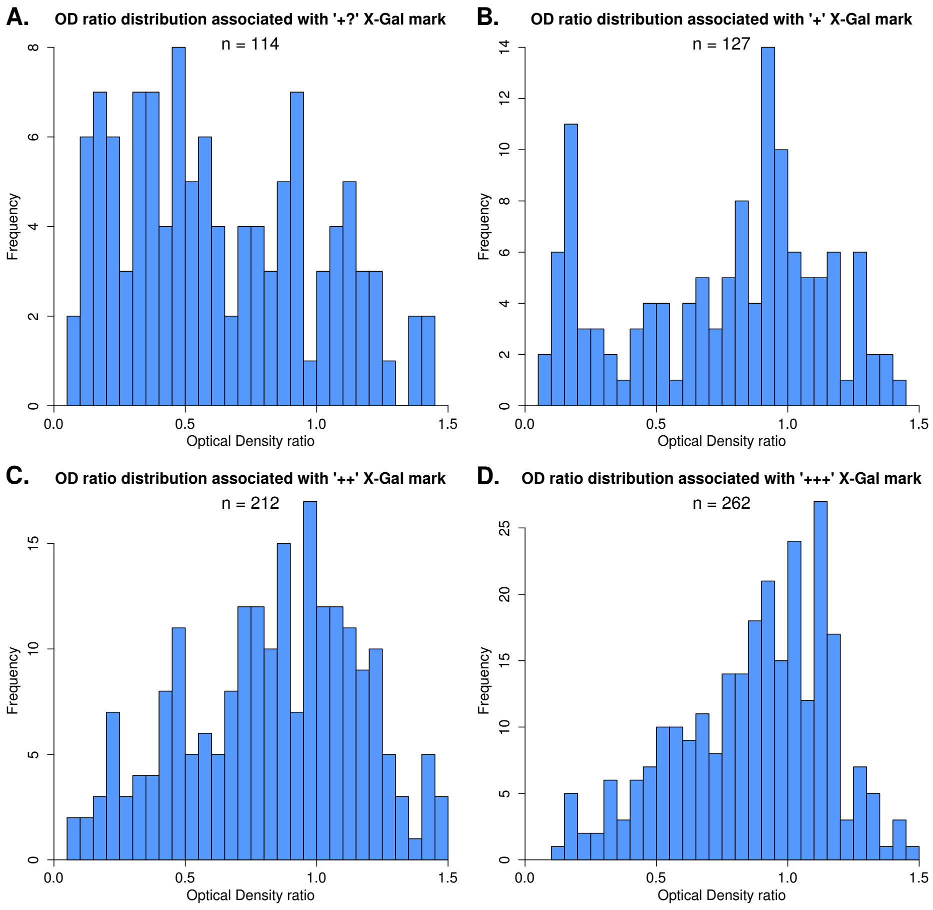

Figure S1 Histograms of optical density ratios (HIS3 test results) for successive X-Gal marks. OD ratios associated to X-Gal marks **(A)** '+?', **(B)** '+', **(C)** '++' and **(D)** '+++'. The OD ratio histogram associated with the X-Gal mark '+' (B) is roughly bimodal. A decrease in the weight of the first component can be observed for the successive higher marks (C and D) until only the second component remains, whose mean is consistently around 1 for the last two marks ('++', '+++'). The first component thus corresponds to 'false positive' and should be discarded using an OD ratio threshold around '0.45' combined with the X-Gal threshold between '+?' and '+'.

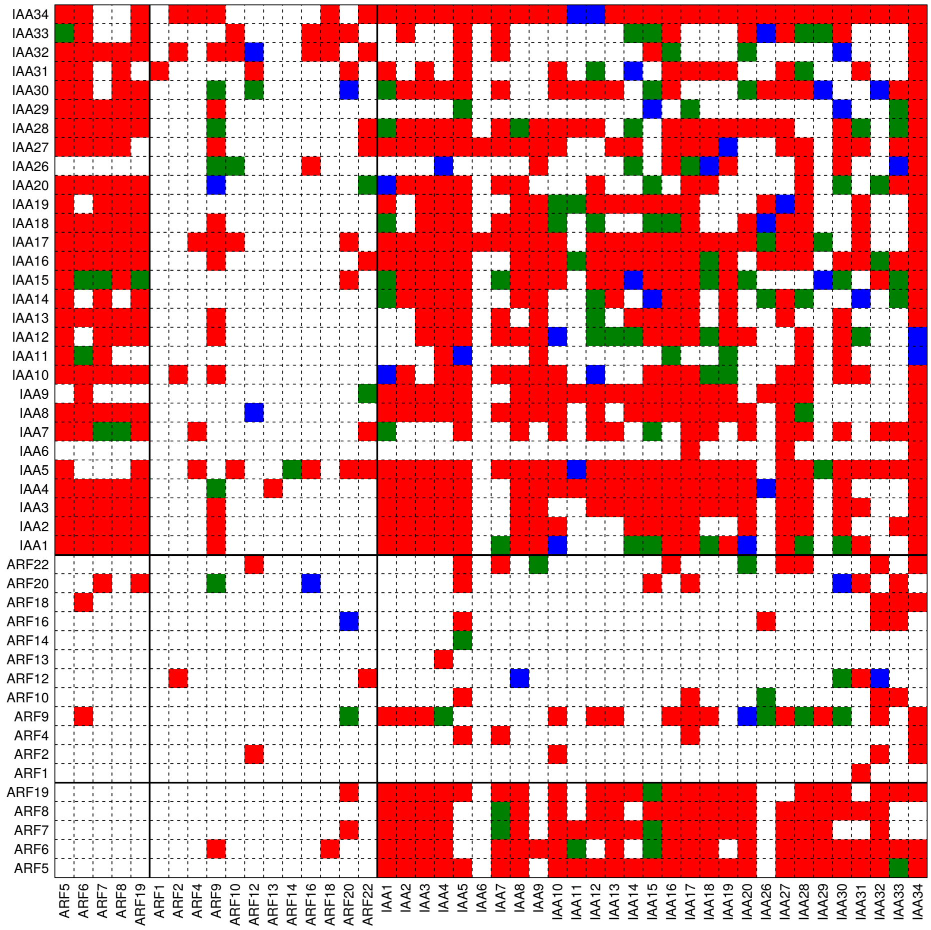

Figure S2 Adjacency matrix obtained using HIS3 thresholds at 0.3, 0.45 and 0.65. The interactions for HIS3 > 0.65 are in red, for HIS3 > 0.45 in red+green and for HIS3 > 0.3 in red+green+blue. The X-Gal threshold is between '+?' and '+'. The transcriptional regulator are grouped by biological families: ARF+, ARF- and Aux/IAA.

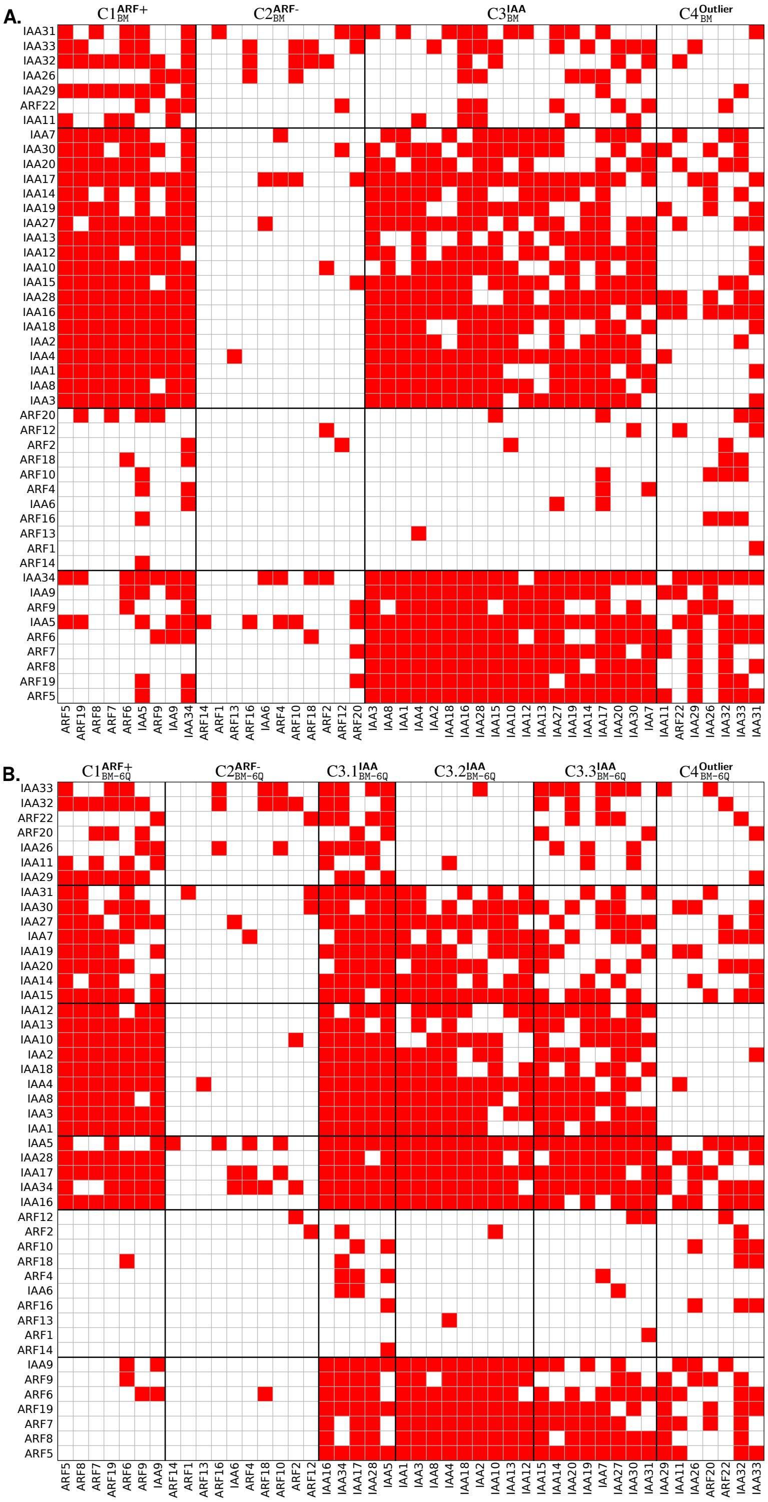

Figure S3 Adjacency matrices with transcriptional regulators grouped by clusters and sorted by increasing within-cluster distances obtained using (A) the 4-cluster and (B) the 6-cluster BM-model. In red are the interaction detected for HIS3 > 0.45 and the X-Gal threshold between '+?' and '+'. **(A)** The 4-cluster BM model present our typical configuration with three biologically meaningful cluster enriched in ARF+ ($C1^{\text{ARF+}}_{\text{BM}}$), ARF- ($C2^{\text{ARF-}}_{\text{BM}}$) and Aux/IAA ($C3^{\text{IAA}}_{\text{BM}}$) and an outlier cluster ($C4^{\text{Outlier}}_{\text{BM}}$). **(B)** The 6-cluster BM model also present a biologically meaningful structure with a more specific enrichment of ARF+ in the first cluster ($C1^{\text{ARF+}}_{\text{BM-6Q}}$), but it splits in three clusters $C3.1^{\text{IAA}}_{\text{BM-6Q}}$, $C3.2^{\text{IAA}}_{\text{BM-6Q}}$ and $C3.3^{\text{IAA}}_{\text{BM-6Q}}$ the Aux/IAA enriched cluster $C3^{\text{IAA}}_{\text{BM}}$. $C3.1^{\text{IAA}}_{\text{BM-6Q}}$ displays a very low dimerisation specificity regarding $C3.2^{\text{IAA}}_{\text{BM-6Q}}$ and $C3.3^{\text{IAA}}_{\text{BM-6Q}}$, while $C3.3^{\text{IAA}}_{\text{BM-6Q}}$ presents a low within-cluster interaction capacity. ARF- enriched and "outlier" cluster compositions are very similar for 4- and 6-cluster BM models.

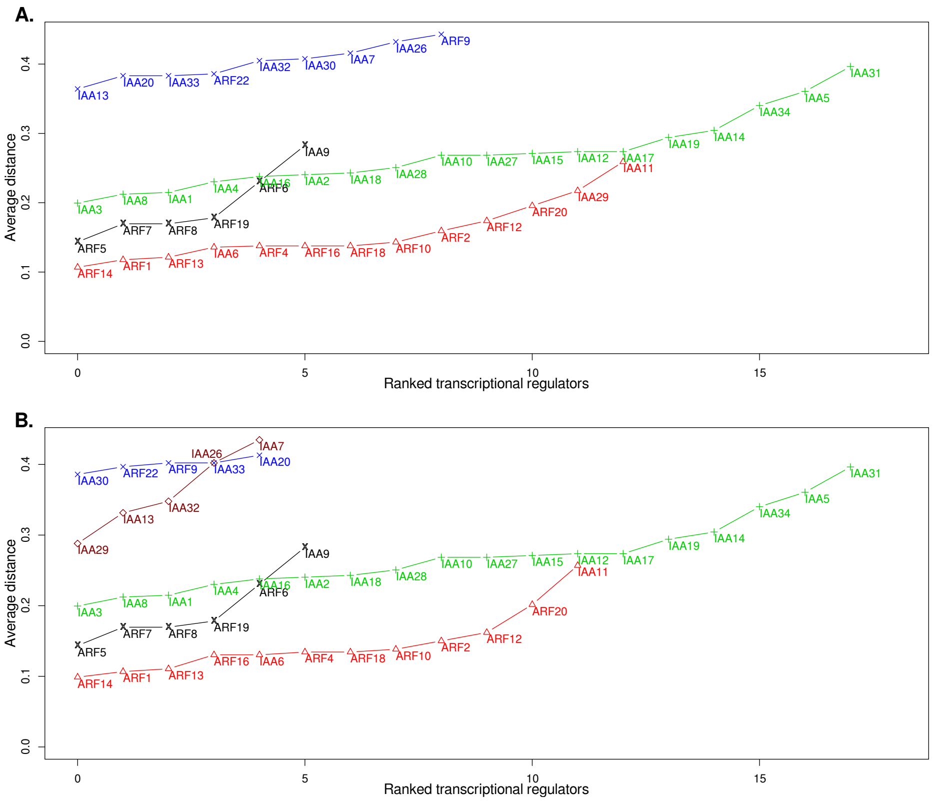

Figure S4 Ranked average distances between each transcriptional regulator and the other transcriptional regulators assigned to the same cluster. **(A)** Clustering obtained using a 4-cluster GM-A model. $C1^{\text{ARF+}}_{\texttt{GM}}$: black; $C2^{\text{ARF-}}_{\texttt{GM}}$: red; $C3^{\text{IAA}}_{\texttt{GM}}$: green; $C4^{\text{Outlier}}_{\texttt{GM}}$: blue. **(B)** Clustering obtained using a 5-cluster GM-A model. $C1^{\text{ARF+}}_{\texttt{GM-5Q}}$: black; $C2^{\text{ARF-}}_{\texttt{GM-5Q}}$: red; $C3^{\text{IAA}}_{\texttt{GM-5Q}}$: green; $C4.1^{\text{Outlier}}_{\texttt{GM-5Q}}$: blue; $C4.2^{\text{Outlier}}_{\texttt{GM-5Q}}$: brown. A close observation shows that the 4- and 5-cluster models are almost perfectly nested. The main difference is the splitting of $C4^{\text{Outlier}}_{\texttt{GM}}$ (blue) into $C4.1^{\text{Outlier}}_{\texttt{GM-5Q}}$ (blue) and $C4.2^{\text{Outlier}}_{\texttt{GM-5Q}}$ (brown). However this splitting does not improve the average distance to other transcriptional regulators of the cluster for the transcriptional regulators assigned to $C4^{\text{Outlier}}_{\texttt{GM}}$ (4-cluster model).

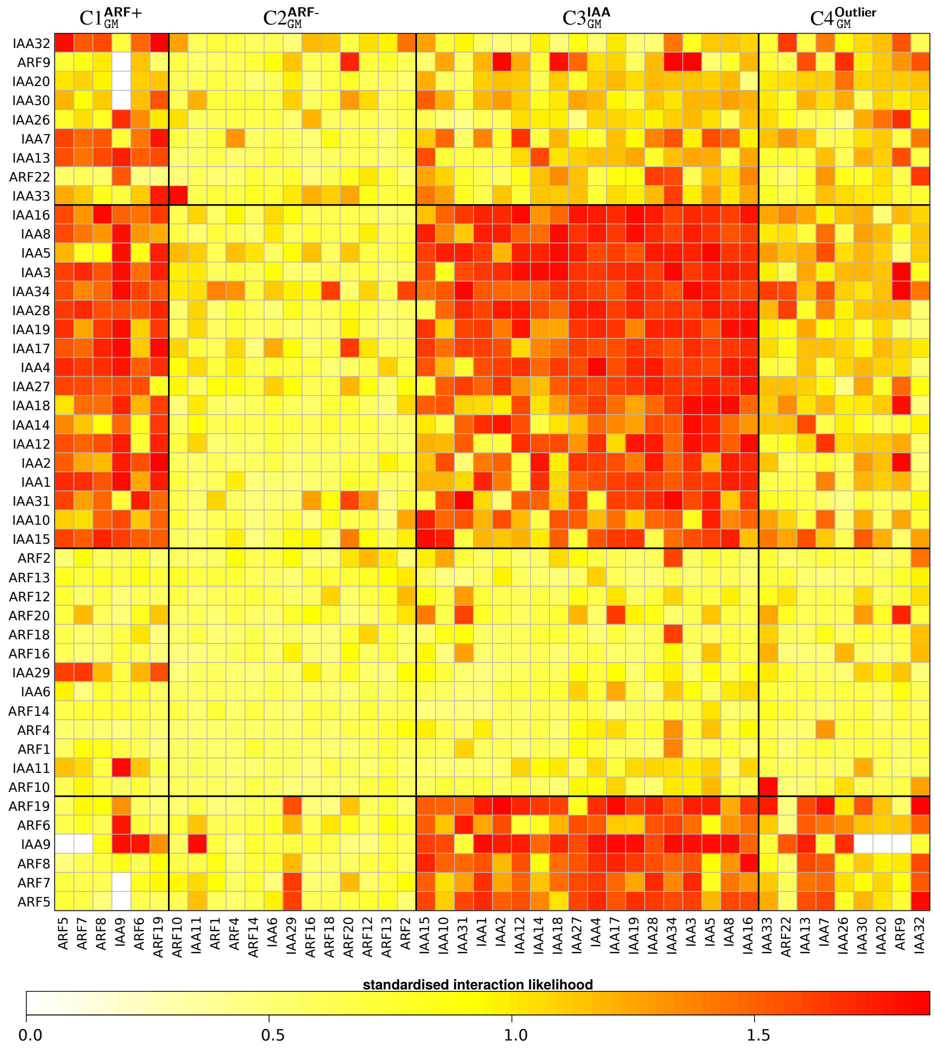

Figure S5 Valued adjacency matrix with transcriptional regulators grouped by clusters and sorted by increasing within-cluster distances obtained using the 4-cluster GM-A model. The standardised interaction likelihoods are given according to a color-scale from white to red. The 4-cluster GM-A model presents our typical configuration with three biologically meaningful cluster enriched respectively in ARF+ ($C1^{\text{ARF+}}_{\text{GM}}$), ARF- ($C2^{\text{ARF-}}_{\text{GM}}$) and Aux/IAA ($C3^{\text{IAA}}_{\text{GM}}$) and an outlier cluster ($C4^{\text{Outlier}}_{\text{GM}}$).

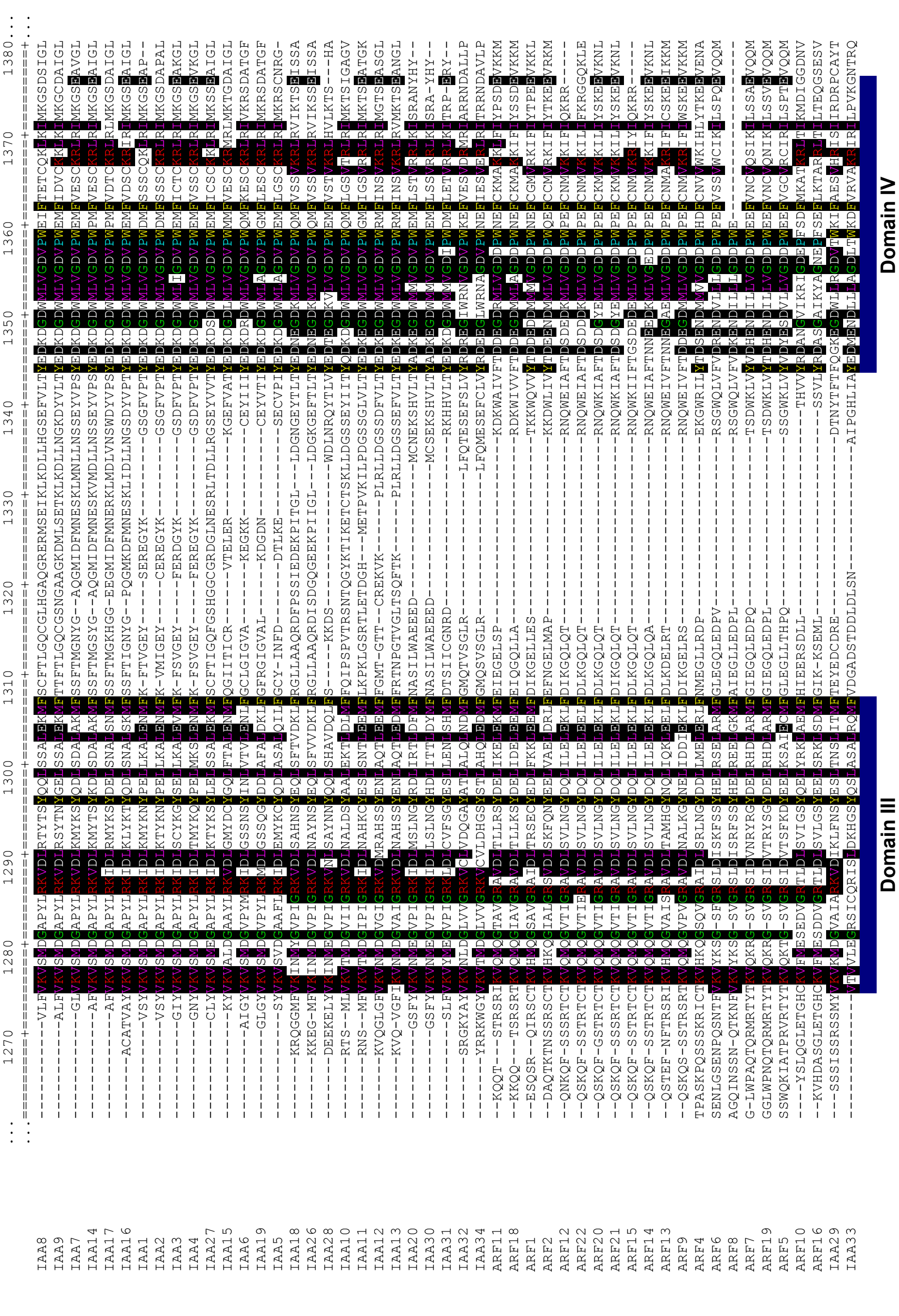

Figure S6 Detail of the multiple alignment curated by Glocks showing domains III and IV. The whole amino-acid sequences of the 49 AGI-accessible Aux/IAA and ARFs have been used to compute the multiple alignment (CLUSTAL-W) and perform the conserved block selection (Glocks). The blue boxes indicate the conserved sequences found by Gblocks. Coloured letters represent highly conserved amino acids. The dashes (-) represent gaps created by the multiple-alignment method to align conserved segments of the sequences.

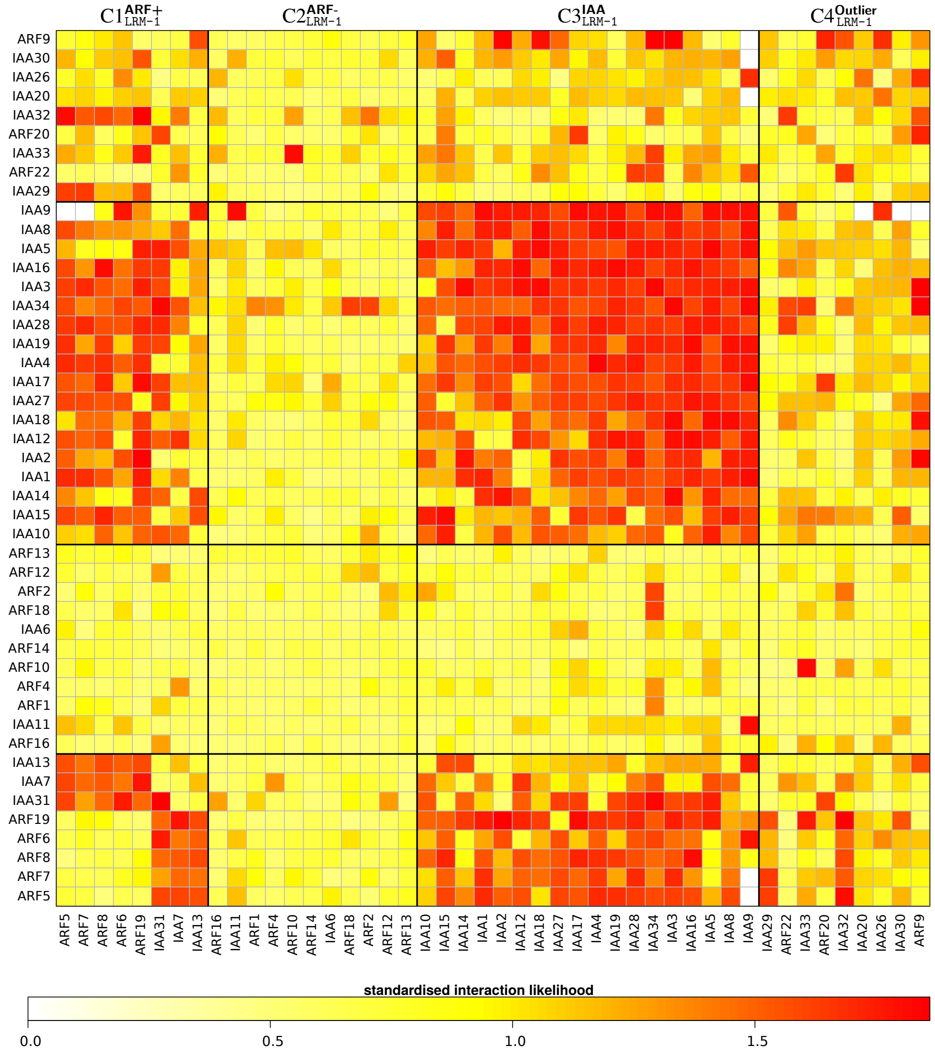

Figure S7 Valued adjacency matrix with transcriptional regulators grouped by clusters and sorted by increasing within-cluster distances obtained using the 4-cluster single-explanatory-variable LRM model. The standardised interaction likelihoods are given according to a color-scale from white to red. The 4-cluster single-explanatory-variable LRM model present our typical configuration with three biologically meaningful cluster enriched respectively in ARF+ ($C1^{\text{ARF+}}_{\text{LRM-1}}$), ARF- ($C2^{\text{ARF-}}_{\text{LRM-1}}$) and Aux/IAA ($C3^{\text{IAA}}_{\text{LRM-1}}$) and an outlier cluster ($C4^{\text{Outlier}}_{\text{LRM-1}}$).

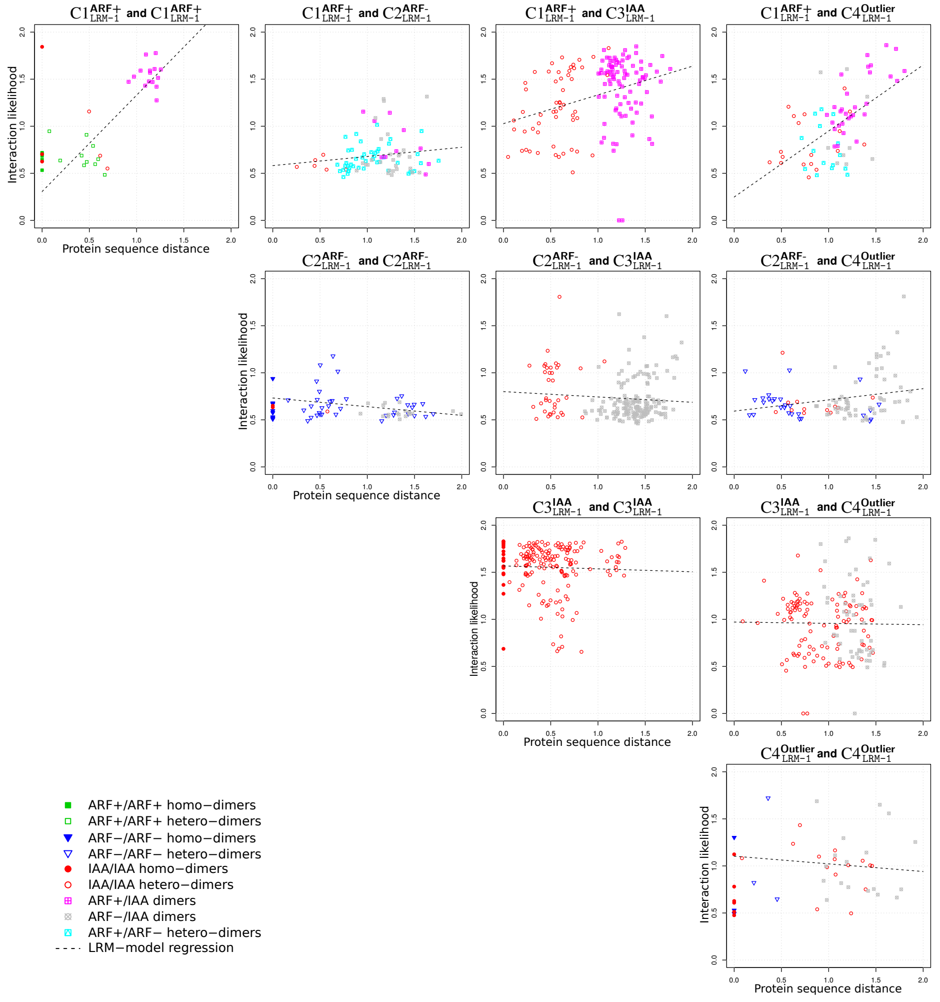

Figure S8 Linear regressions for each pair of clusters within the 4-cluster single-explanatory-variable LRM model. The outlier cluster is also represented. The nine possible dimer configurations have been mapped according to the three sub-families ARF+, ARF- and Aux/IAA. The dashed lines represent the estimated linear relationship between the protein sequence distances and the interaction likelihoods for each pair of clusters.

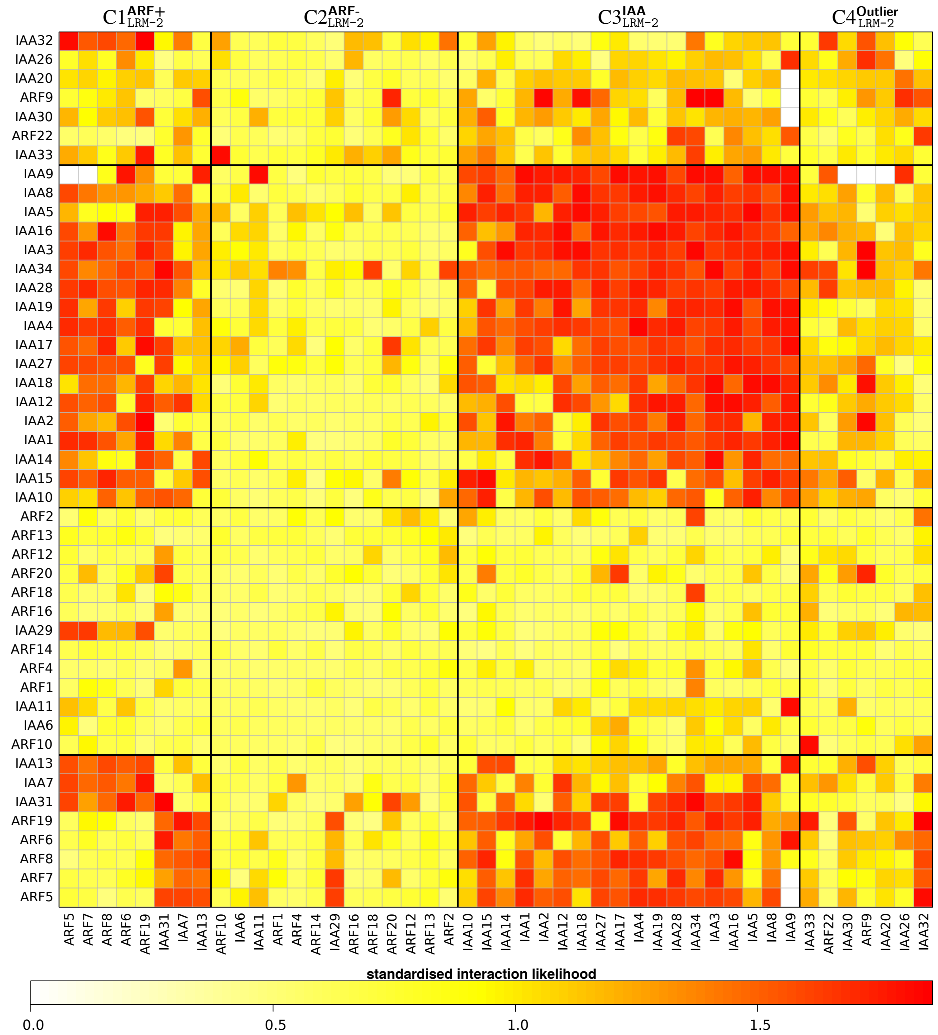

Figure S9 Valued adjacency matrix with transcriptional regulators grouped by clusters and sorted by increasing within-cluster distances obtained using the 4-cluster two-explanatory-variable LRM model. The standardised interaction likelihoods are given according to a color-scale from white to red. The 4-cluster two-explanatory-variable LRM model present our typical configuration with three biologically meaningful cluster enriched respectively in ARF+ ($C1^{\text{ARF+}}_{\text{LRM-2}}$), ARF- ($C2^{\text{ARF-}}_{\text{LRM-2}}$) and Aux/IAA ($C3^{\text{IAA}}_{\text{LRM-2}}$) and an outlier cluster ($C4^{\text{Outlier}}_{\text{LRM-2}}$).

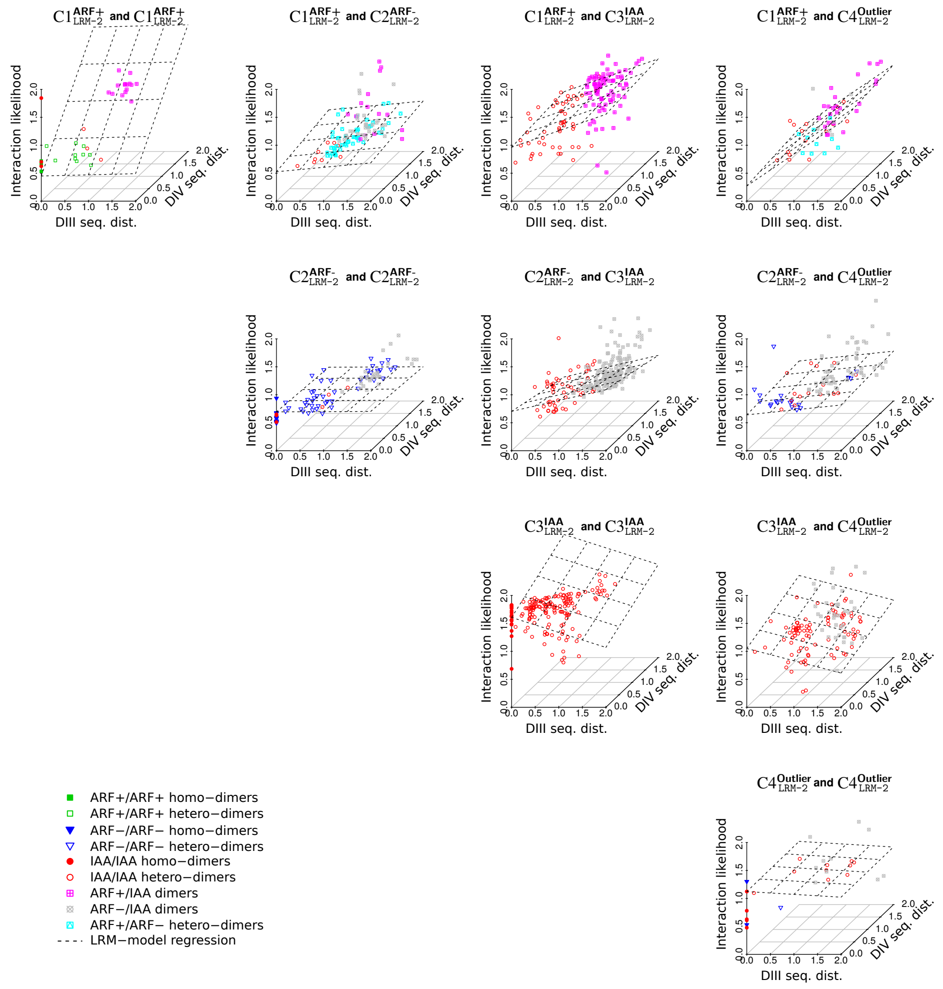

Figure S10 Linear regressions for each pair of clusters within the 4-cluster two-explanatory-variable LRM model. The outlier cluster is also represented. The nine possible dimer configurations have been mapped according to the three sub-families ARF+, ARF- and Aux/IAA. The dashed planes represent the domain-specific estimated linear relationships between the protein sequence distances and the interaction likelihoods for each pair of clusters.

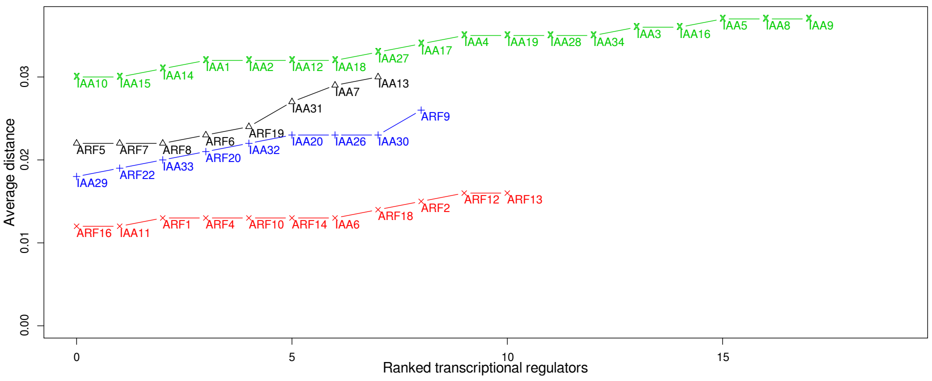

Figure S11 Ranked average distances between each transcriptional regulator and the other transcriptional regulators assigned to the same cluster. Clustering obtained using a 4-cluster single-explanatory-variable LRM model.

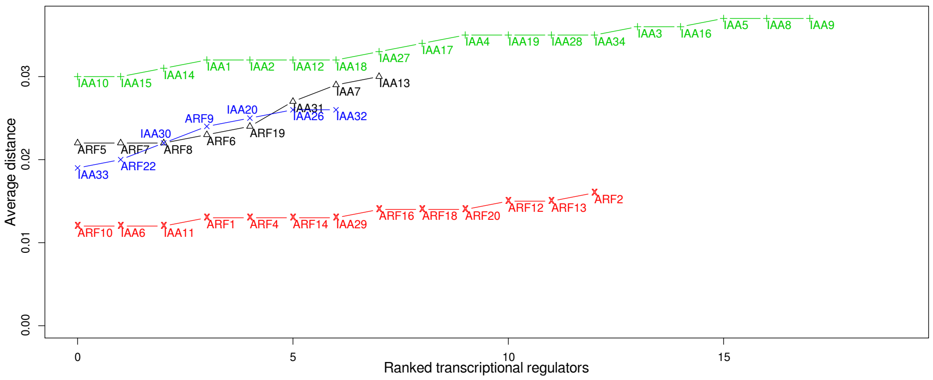

Figure S12 Ranked average distances between each transcriptional regulator and the other transcriptional regulators assigned to the same cluster. Clustering obtained using a 4-cluster two-explanatory-variable LRM model.
