# Supplementary material for: Modelling the influence of dimerisation sequence dissimilarities on the auxin signalling network
Source: BMC Syst Biol. 2016 Mar 1;10:22. doi: 10.1186/s12918-016-0254-7 (PMC4774195; doi:10.1186/s12918-016-0254-7)
Supplement: Additional file 2 — Supplemental Tables. This file contains between-cluster distance matrices for the 6-cluster BM model in Table S1. and the 5-cluster GM-A model in Table S2. (PDF 75.2 kb) [file 12918_2016_254_MOESM2_ESM.pdf]

Additional file 2 — Supplemental Tables.

Table S1 Between-cluster distance matrix $D(q, \ell)$ obtained using the 6-cluster BM model.

$$D_{\text{BM}}(q,\ell) = \begin{array}{cccccc} C1^{\text{ARF+}}_{\texttt{BM-6Q}} & C2^{\text{ARF-}}_{\texttt{BM-6Q}} & C3.1^{\text{IAA}}_{\texttt{BM-6Q}} & C3.2^{\text{IAA2}}_{\texttt{BM-6Q}} & C3.3^{\text{IAA}}_{\texttt{BM-6Q}} & C4^{\text{Outlier}}_{\texttt{BM-6Q}} \end{array} \\ \begin{pmatrix} 0.213 & 0.484 & 0.343 & 0.367 & 0.370 & 0.513 \\ 0.484 & 0.110 & 0.673 & 0.525 & 0.476 & 0.280 \\ 0.343 & 0.673 & 0.257 & 0.292 & 0.351 & 0.561 \\ 0.367 & 0.525 & 0.292 & 0.179 & 0.295 & 0.408 \\ 0.370 & 0.476 & 0.351 & 0.295 & 0.333 & 0.447 \\ 0.513 & 0.280 & 0.561 & 0.408 & 0.447 & 0.323 \end{pmatrix}$$

Table S2 Between-cluster distance matrix $D(q, \ell)$ obtained using the 5-cluster GM-A model.

$$D_{\text{GM-A}}(q,\ell) = \begin{array}{ccccc} C1^{\text{ARF+}}_{\texttt{GM-A-5Q}} & C2^{\text{ARF-}}_{\texttt{GM-A-5Q}} & C3^{\text{IAA}}_{\texttt{GM-A-5Q}} & C4.1^{\text{Outlier}}_{\texttt{GM-A-5Q}} & C4.2^{\text{Outlier}}_{\texttt{GM-A-5Q}} \end{array} \\ \begin{pmatrix} 0.015 & 0.015 & 0.032 & 0.019 & 0.030 \\ 0.015 & 0.014 & 0.016 & 0.016 & 0.015 \\ 0.032 & 0.016 & 0.033 & 0.023 & 0.020 \\ 0.019 & 0.016 & 0.023 & 0.020 & 0.024 \\ 0.030 & 0.015 & 0.020 & 0.024 & 0.016 \end{pmatrix}$$
